# Steroidal Alkaloids from *Sarcococca saligna* (Buxaceae): In Vitro and In Silico Evaluation of Their Cytotoxic Potential

**DOI:** 10.1021/acsomega.5c04143

**Published:** 2025-10-10

**Authors:** Neha Sahu, Amit Dubey, Nitesh Singh, K.R. Arya, Pragya Yadav, Priyank Chaturvedi, Sanjeev Meena, Vijaya Shukla, Dipak Datta, Narender Tadigoppula, Brijesh Kumar, Bikash Kumar Rajak

**Affiliations:** † Department of Botany, 30096 University of Lucknow, Lucknow, Uttar Pradesh 226031, India; ‡ Center for Global Health Research, Saveetha Medical College and Hospitals, 194347 Saveetha Institute of Medical and Technical Sciences, Chennai, Tamil Nadu 600077, India; § Department of Plant Protection, Faculty of Agricultural Sciences, 389839 SGT University, Gurugram, Haryana 122505, India; ∥ Botany Division, CSIR-Central Drug Research Institute, Lucknow, Uttar Pradesh 226031, India; ⊥ Medicinal and Process Chemistry Division, 30082 CSIR-Central Drug Research Institute, Lucknow, Uttar Pradesh, 226031 India; # Biochemistry, CSIR-Central Drug Research Institute, Lucknow, Uttar Pradesh 226031, India; ∇ Sophisticated Analytical Instrument Facilities, 30085 CSIR-Central Drug Research Institute, Lucknow, Uttar Pradesh 226031, India; ○ Department of Bioinformatics, School of Earth, Biological and Environmental Sciences, 206411 Central University of South Bihar, Gaya, Bihar 824236, India

## Abstract

*Sarcococca saligna* (*S. saligna*), a medicinal shrub rich in therapeutic steroidal alkaloids (SAs), is ethnomedicinally used to treat ulcers, tumors, and wounds. In this study, to explore the anticancer potential of *S. saligna*, a combination of bioactivity-guided fractionation and isolation, an in vitro cytotoxic assay, and in silico analysis was used. The ethanolic extract of *S. saligna* (SL-01) was fractionated into butanol (SL-02), ethyl acetate (SL-03), hexane (SL-04), and water (SL-05) fractions. The extract and fractions, along with isolated compounds, were tested for anticancer activity against human cancer cell lines (colon, lung, and breast) using the sulforhodamine assay, with SL-03 displaying the strongest cytotoxicity in HT-29 colon cancer cells (IC_50_ = 18.6 μM). The most active fraction SL-03 confirmed the presence of eight bioactive steroidal alkaloids *via* LC-ESI-QTOF-MS/MS analysis. Two SAs sarcorine C and salonine C isolated from SL-03 were structurally confirmed through NMR spectroscopy and exhibited selective cytotoxicity against HT-29 cells with minimal activity in noncancerous cell lines. The markedly lower IC_50_ values of salonine C (5.21 μM) and sarcorine C (3.25 μM) highlight their potential as safer, more effective lead candidates for colon cancer therapy. Computational pharmacokinetics (SwissADME, ADMET analysis) predicted favorable drug-likeness, and DFT calculations provided electronic characteristics for both compounds. Moreover, molecular docking of both compounds with key cancer-associated targets CDK2, CYP17A1, Bcl-2, and MMP-2 showed stable binding. Additionally, extended 200 ns molecular dynamics simulations further validated the complexes, revealing stable RMSD, reduced SASA, favorable hydrogen bonding, and strong MM-GBSA binding free energies (△G_bind = −42.6 kcal·mol^–1^ for sarcorine C vs −40.8 kcal·mol^–1^ for roscovitine). These findings establish *S. saligna* as a promising source of anticancer steroidal alkaloids and report, for the first time, the selective cytotoxic activity of sarcorine C and salonine C against colon cancer cells, supported by integrated experimental and computational evidence.

## Graphical abstract

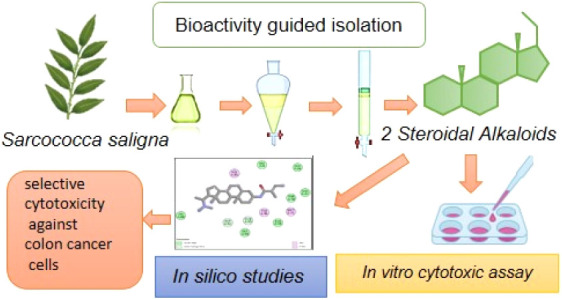

## 1 Introduction

Cancer remains one of the leading causes of mortality worldwide, with an estimated 19.3 million new cases and nearly 10 million deaths reported in 2020.^1,2^ Current therapeutic strategies, including surgery, radiotherapy, immunotherapy, and chemotherapy, are limited by high costs, toxicity, or the development of resistance.^3–6^ Chemotherapeutic drugs can temporarily suppress tumor progression, but prolonged treatment often results in multidrug resistance, tumor recurrence, systemic toxicity, and weakened immunity.^7^ This highlights the urgent need for safer, affordable, and effective alternatives, either as standalone therapies or in combination with existing regimens.

Natural products, particularly phytochemicals, have historically contributed to anticancer drug discovery. Several clinically used agents, such as vincristine, etoposide, irinotecan, and paclitaxel, are of plant origin.^8–10^ Recently, global interest in phytopharmaceuticals has increased, and in India, amendments to the Drugs and Cosmetics Act have promoted the development of botanical-based drugs.^11,12^ Among natural metabolites, steroidal alkaloids have attracted particular attention due to their dual steroidal and alkaloid characteristics, which confer significant biological activity, including cytotoxic, immunomodulatory, and antiacetylcholinesterase effects.^13–15^

*Sarcococca saligna* (Buxaceae), locally known as Piruli, Geru, or Tiliara, is an evergreen shrub widely distributed across South and Southeast Asia, including the Himalayas, Afghanistan, Nepal, and Sri Lanka.^16,17^ Ethnomedicinally, *S. saligna* has been used to treat ulcers, tumors, joint pain, arthritis, and diabetes and exhibits diverse pharmacological activities such as antiulcer, hepatoprotective, anti-inflammatory, antibacterial, antifungal, and cytotoxic properties.^18–24^ Phytochemical investigations have identified steroidal alkaloids as the principal bioactive constituents of this plant, predominantly pregnane-type (C-21) alkaloids.^25–27^ These compounds are of particular interest due to their reported cytotoxic and anticancer-related activities.

In view of its rich ethnomedicinal background and phytochemistry, this study aimed to evaluate the cytotoxic potential of *S. saligna* extracts, fractions, and isolated compounds (Figure 1) against human cancer cell lines (HT-29, A549, MCF-7, and DLD-1) using sulforhodamine (SRB) assay. HT-29 and DLD-1 are human colorectal adenocarcinoma cell lines, A549 is a human lung carcinoma epithelial cell line, and MCF-7 is an estrogen receptor–positive human breast adenocarcinoma cell line. To gain mechanistic insights, experimental assays were complemented with computational approaches, such as ADMET predictions (absorption, distribution, metabolism, excretion, and toxicity), density functional theory (DFT) calculations, molecular docking, and molecular dynamics simulations. Accordingly, six cancer-relevant proteins were selected owing to their critical roles in cell cycle regulation, apoptosis, angiogenesis, and metastasis. Cyclin-dependent kinase 2 (CDK2, PDB: 2A4L), cytochrome P450 CYP17A1 (CYP17A1, PDB: 3RUK), B-cell lymphoma 2 (Bcl-2, PDB: 4LXD), matrix metalloproteinase-2 (MMP-2, PDB: 1HOV), nitric oxide synthase (NOS, PDB: 3E7G), and Kirsten rat sarcoma viral oncogene homologue (KRAS, PDB: 4LPK) were selected as target proteins for molecular docking (Table 1). This integrated framework provides the first report of sarcorine C and salonine C as selective cytotoxic agents against HT-29 colon cancer cells, reinforcing *S. saligna* as a promising source of anticancer phytochemicals.

**Figure 1:**
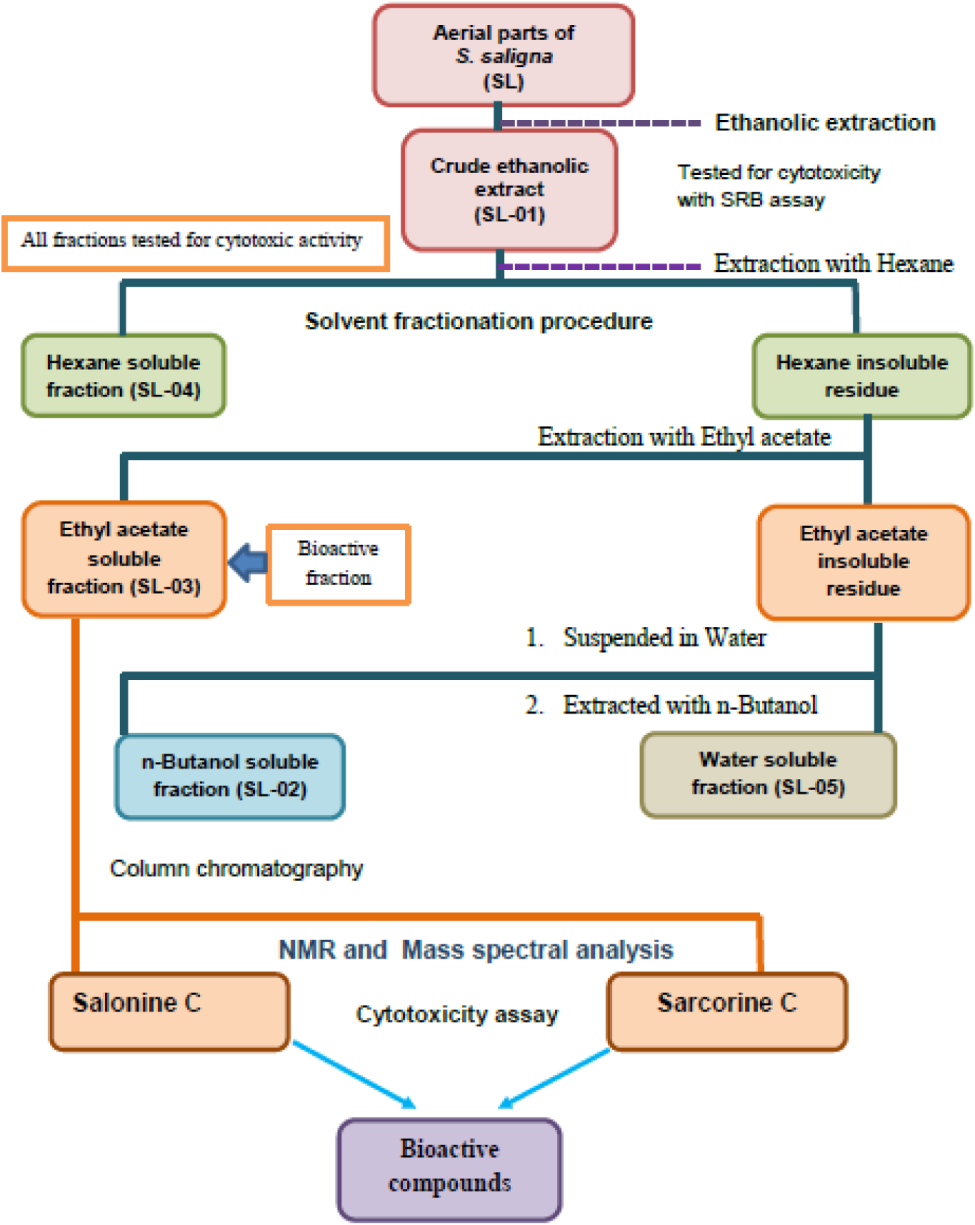
Schematic presentation of extraction and solvent fractionation of *Sarcococca saligna*.

**Table 1:** PDB (Protein Data Bank) IDs of Target Proteins

PDB ID	Protein Full Name	Abbreviation
2A4L	Cyclin-dependent kinase 2	CDK2
3RUK	Cytochrome P450 CYP17A1	CYP17A1
4LXD	Apoptosis regulator Bcl-2	Bcl-2
1HOV	Matrix metalloproteinase-2	MMP-2
3E7G	Nitric oxide synthase	NOS
4LPK	Kirsten rat sarcoma viral oncogene	KRAS

## 2 Results and Discussion

### 2.1 In Vitro Cytotoxicity of Ethanolic Extract and Its Different Solvent Fractions

#### 2.1.1 Percentage Cell Growth Inhibition

Preliminary cytotoxicity screening of the ethanolic extract (SL-01) against cancer cell lines, viz., DLD-1, MCF-7, A-549, and HT-29, showed considerable cytotoxicity with % cell growth inhibition (GI) of 57.42, 56.99, 88.78, and 48.82%, respectively (Table 2). Moreover, this value was also notably high against noncancerous monkey kidney fibroblast cell lines (VERO cell line), with 56.36% cell GI. The ethanolic extract demonstrated promising cytotoxicity across the tested cancer cell lines, with the highest activity observed against A-549 lung cancer cells, suggesting its potential as a broad-spectrum anticancer agent due to the presence of polar and moderately nonpolar compounds.

**Table 2:** Effect of Ethanolic Extract (SL-01) and Their Different Solvent Fractions on Percent Growth Inhibition of Different Human Cancer Cell Lines at 100 μg/mL Concentration

	Cancer cell lines				Noncancerous cell line
Crude and fractions^a^	DLD-1	MCF-7	A-549	HT-29	VERO
**SL-01**	57.42 ± 0.3	56.99 ± 0.02	88.78 ± 1.1	48.82 ± 1.5	56.36 ± 0.12
**SL-02**	74.03 ± 4.6	28.99 ± 0.17	55.54 ± 1.02	60.70 ± 0.9	5.69 ± 2.8
**SL-03**	91.52 ± 1.1	70.81 ± 0.87	88.94 ± 1.13	86.25 ± 1.1	55.96 ± 0.11
**SL-04**	47.34 ± 0.8	40.81 ± 1.5	41.87 ± 1.76	4.97 ± 0.22	13.29 ± 0.33
**SL-05**	15.57 ± 1.4	-2.454 ± 0.6	9.90 ± 1.17	9.9 ± 0.46	–10.27 ± 1.23

a Ethanolic extract (SL-01); *n*-butanol (SL-02); ethyl acetate (SL-03); hexane (SL-04); water (SL-05).

Four solvent fractions were subsequently tested against the same panel of cell lines, where the ethyl acetate fraction showed the highest activity, with consistent cytotoxicity, i.e., 91.52, 70.81, 88.94, and 86.25% GI against DLD-1, MCF-7, A-549, and HT-29 cell lines, respectively (Table 2). However, the GI against the noncancerous VERO cell line was also very high (55.96%). Interestingly, SL-02 was also found to be effective against the DLD-1 cell line, with 74.03% cell GI, while exhibiting comparatively lower toxicity toward the VERO cell line. However, in this study, we focused only on the highest bioactive fraction SL-03 and proceeded with it for further exploration. Ethyl acetate fractions of *Sarcococca* species are rich in steroidal alkaloids, which are well-documented for their cytotoxic effects.^22,28–30^ These compounds induce apoptosis and inhibit cell proliferation by targeting critical cancer pathways like p53 activation, mitochondrial membrane disruption, and inhibition of topoisomerase enzymes.^28–30^

#### 2.1.2 IC_50_ Value of SL-03 Bioactive Fraction

To assess the cell-specific cytotoxic selectivity of the SL-03 fraction, dose-dependent studies (6.25–100 μg/mL) were performed against the same panel of cell lines (Figure 2A–D).

**Figure 2:**
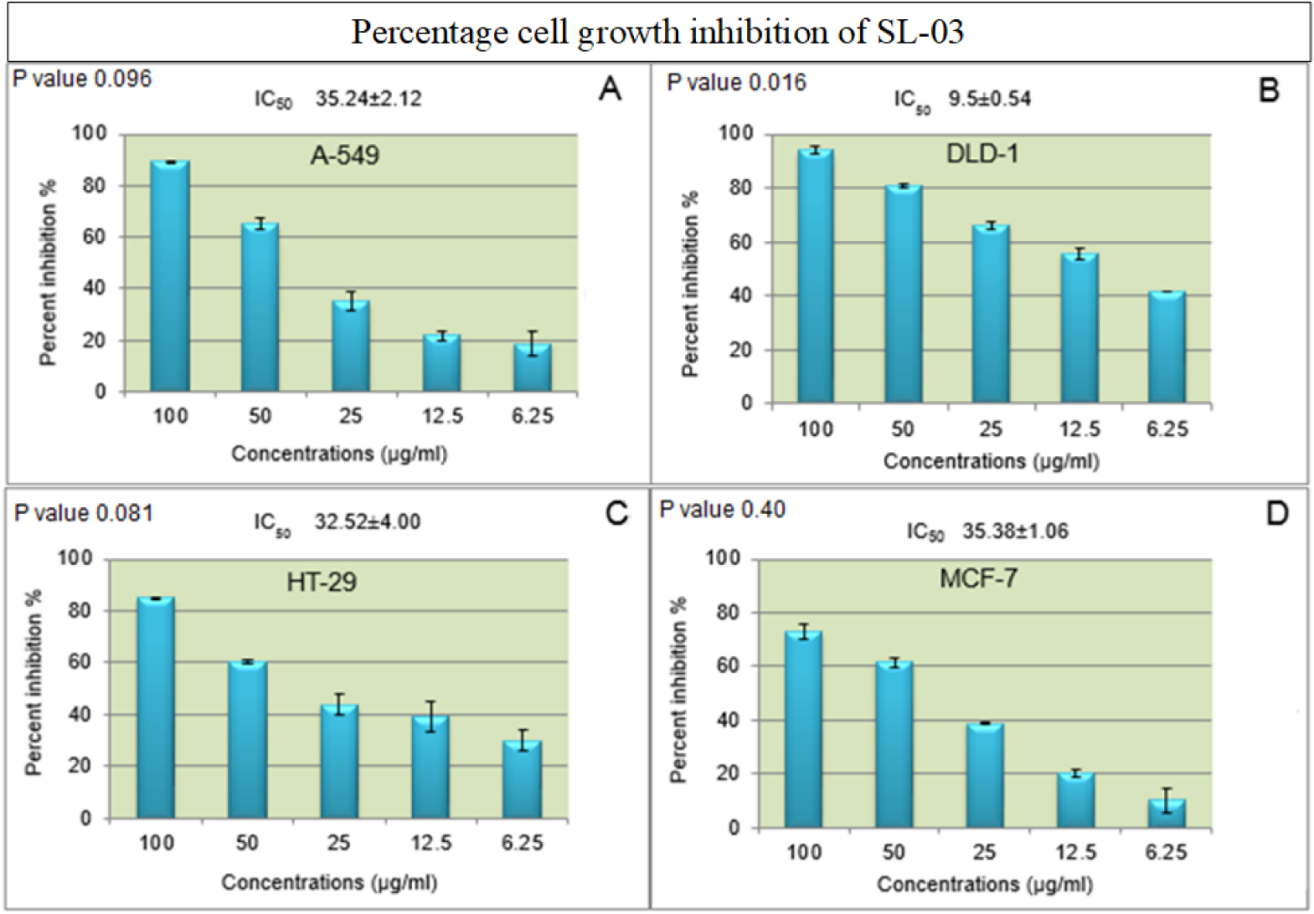
(A–D) Percent growth inhibition and determination of IC_50_ values of the SL-03 bioactive fraction against A549, DLD-1, HT-29, and MCF-7 at different concentrations.

Results revealed that the SL-03 fraction exhibited the highest cytotoxic susceptibility toward the DLD-1 cell line, with the lowest IC_50_ value of 9.5 μg/mL, followed by 32.52 μg/mL in HT-29 and 35.24 μg/mL in A-549 cells (Figure 2A–D). In MCF-7 cells, SL-03 exhibited 70% cytotoxicity with an IC_50_ value of 35.38 μg/mL. Comparison of IC_50_ values among tested cell lines indicates that the SL-03 fraction was most effective against DLD-1 and HT-29 cell lines, as indicated by their lowest IC_50_ values, suggesting selective cytotoxicity toward colorectal cancer cells (Figure 2A–D).

However, SL-03 also exhibited considerable cytotoxicity (55.96% GI) against the noncancerous VERO cell line (Table 2), indicating limited selectivity at the tested concentrations and suggesting the need for further purification through bioactivity-guided isolation to enhance its therapeutic index. The strong cytotoxicity of SL-03 toward DLD-1 and HT-29 cells may reflect its targeted action on pathways specific to colon cancer, such as apoptosis induction *via* mitochondrial disruption or caspase activation, and cell-cycle arrest at the G2/M phase, as previously reported for related fractions.^31^ Furthermore, differences in IC_50_ values across cell lines suggest that the active compounds in SL-03 may preferentially target molecular pathways or receptors highly expressed in colon cancer cells, thereby explaining its enhanced potency against DLD-1 and HT-29. These findings prompted further phytochemical investigation of the SL-03 fraction, leading to the isolation of 2 major bioactive compounds.

### 2.2 Phytochemical Profiling of Bioactive Fraction

#### 2.2.1 LC-ESI-QTOF-MS/MS Analysis of SL-03

The base peak chromatogram (BPC) and Liquid chromatography–electrospray ionization–quadrupole time-of-flight–tandem mass spectrometry (LC-ESI-QTOF-MS/MS) profiles of the bioactive fraction SL-03 are shown in Figure 3 and Table 3. The LC-ESI-QTOF-MS/MS profile enabled the identification of eight steroidal alkaloids namely axillarine F, warsine A, sarcorine C, axillarine C, salonine C, vaganine A, salignenamide A, and axillaridine A. Among these, axillaridine A was confirmed using its corresponding authenticated standard, while the remaining seven compounds were tentatively identified based on their MS/MS fragmentation patterns (data not shown) and comparison with previously reported literature.^19,28^

**Table 3:** LC-ESI-QTOF-MS/MS Analysis of Bioactive Ethyl Acetate Fraction (SL-03)

S.N.	Chemical formula	Calculated mass [M + H]^+^	Measured mass [M + H]^+^	Error (Δppm)	*T*_R(a)_	*T*_R(b)_	Detected compounds^a^
1	C_30_H_50_N_2_O_4_	503.3843	503.3847	0.78	7.9	NA	Axillarine F
2	C_28_H_46_N_2_O_2_	443.3632	443.3628	0.87	8.4	NA	Warsine A
3	C_25_H_44_N_2_O	389.3526	389.3521	1.9	8.6	NA	Sarcorine C
4	C_32_H_48_N_2_O_4_	525.3687	525.3682	0.74	8.7	NA	Axillarine C
5	C_28_H_44_N_2_O	425.3526	425.3524	0.5	12	NA	Salonine C
6	C_30_H_50_N_2_O_3_	487.3894	487.389	1.24	12.2	NA	Vaganine A
7	C_30_H_52_N_2_O	457.4152	457.4158	1.61	17.4	NA	Salignenamide A
8	C_30_H_42_N_2_O_2_	463.3319	463.3315	0.81	18	17.9	Axillaridine A

a *T*_R(a)_: retention time of the **isolated compound** in chromatogram. *T*_R(b)_: retention time of the **standard** compound in chromatogram. All the 8 compounds are steroidal alkaloids; NA: not available.

**Figure 3:**
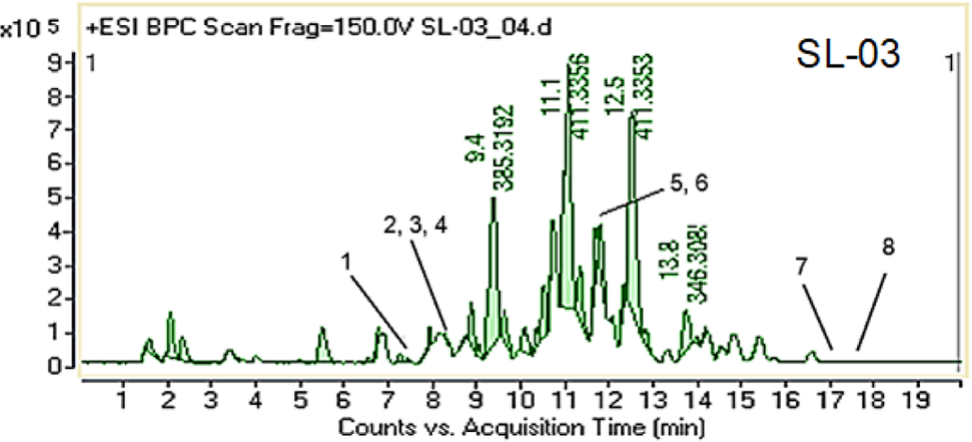
Base peak chromatogram (BPC) of the SL-03 fraction showing eight identified steroidal alkaloids. Retention times (min): 1. axillarine F (7.9), 2. warsine A (8.4), 3. sarcorine (8.6), 4. axillarine C (8.7), 5. salonine C (12), 6. vaganine A (12.2), 7. salignenamide A (17.4), and 8. axillaridine A (18).

### 2.3 Molecular Docking of All 8 Compounds against Key Regulators of Cancer

Docking data revealed that axillarine C exhibited the strongest binding affinity, with −6.5 kcal/mol against CYP17A1 and −6.4 kcal/mol against NOS . To validate the docking protocol, reference inhibitors for each protein were redocked into their respective binding sites. RMSD (Root Mean Square Deviation) values of <2.0 Å confirmed docking accuracy, while their binding energies provided benchmarks for comparison with the steroidal alkaloids. Notably, sarcorine C exhibited a binding affinity close to that of the CDK2 inhibitor roscovitine, while axillarine C showed favorable interactions with CYP17A1 and NOS, underscoring their potential as selective anticancer leads (Table 4). These results identify axillarine C as a promising anticancer candidate.^32–37^ **Salonine C** displayed moderate to strong binding across the selected receptors, with docking scores ranging from −4.4 to −5.6 kcal/mol. The strongest interaction was observed with NOS (−5.6), followed by MMP-2 (−5.3) and CYP17A1 (−5.3), indicating promising stability of interactions of salonine C with cancer-related enzymes.^38–41^ Saligenamide A and axillaridine also demonstrated strong binding interactions, supporting their relevance for further investigation. In contrast, warsine A, sarcorine C, and axillarine F displayed only moderate affinities, whereas vaganine A exhibited the weakest binding, particularly with NOS. Overall, these results highlight axillarine C as the lead compound, with saligenamide A and axillaridine as supportive candidates for future validation studies (Table 4).

**Table 4:** Molecular Docking Results of Eight Steroidal Alkaloids from *Sarcococca saligna* and Reference Compounds against Key Cancer-Associated Targets

Target Protein (PDB ID)	Reference Ligand (FDA/known inhibitor)KI	Docking Score (kcal/mol)	RMSD (Å)	Best Steroidal Alkaloid (Docking Score, kcal/mol)	Comparative Remarks
CDK2 (2A4L)	Roscovitine	–8.2	1.42	Sarcorine C (−5.6), salonine C (−5.3)	Alkaloids show moderate affinity; sarcorine C forms stable H-bonds (Asp86, Gln131), supported by MD/MMGBSA.
CYP17A1 (3RUK)	Abiraterone	–9.1	1.63	Axillarine C (−6.5), salonine C (−5.3)	Axillarine C positioned near heme group, mimicking abiraterone’s catalytic inhibition.
Bcl-2 (4LXD)	Venetoclax	–10.4	1.58	Axillaridine A (−6.1), salonine C (−5.0)	Steroidal alkaloids engage BH3 groove, but weaker than venetoclax.
MMP-2 (1HOV)	Marimastat	–9.5	1.37	Salonine C (−5.3), sarcorine (−5.0)	Salonine C interacts with catalytic Zn^2+^ site, supporting antimetastatic potential.
NOS (3E7G)	L-NAME	–7.8	1.29	Axillarine C (−6.4), salonine C (−5.6)	Comparable binding modes observed; alkaloids stabilize *via* electrostatic interactions (Arg199, Glu377).
KRAS (4LPK)	AMG 510 (sotorasib)	–8.5	1.88	Sarcorine C (−4.7), salonine C (−4.4)	Moderate binding; noncovalent interactions suggest alkaloids may weakly modulate KRAS activity.

#### 2.3.1 Molecular Docking of Axillarine C against Key Cancer Targets

The receptors selected for docking of the eight bioactive compounds were CDK2, CYP17A1, Bcl-2, MMP-2, and NOS (Table 1). These represent critical regulators of the cell cycle, apoptosis, metastasis, and tumor signaling.^38–40^ CDK2 is a cyclin-dependent kinase that regulates the G1-S phase transition and is frequently targeted in cancer therapy.^38^ Several CDK inhibitors are in clinical trials, reinforcing their therapeutic importance. CYP17A1 is a steroidogenic cytochrome P450 enzyme crucial for androgen and estrogen biosynthesis.^39^ Its inhibition forms the basis of abiraterone therapy in prostate cancer. Bcl-2 is an antiapoptotic protein whose overexpression not only enhances cancer cell survival but also contributes to resistance against chemotherapeutic agents, thereby representing a critical target for overcoming drug resistance in cancer therapy.^40^ MMP-2 is a matrix metalloproteinase that degrades extracellular matrix components, thereby promoting tumor invasion and metastasis; its overexpression has been strongly associated with increased invasiveness and poor prognosis in colon and lung cancers.^41^ NOS produces nitric oxide, a signaling molecule involved in vascular, neural, and immune regulation, and its dysregulation contributes to tumor angiogenesis and progression.^42^ The in silico analysis revealed that axillarine C exhibited strong binding affinities across all five target proteins, with binding energies up to −6.5 kcal/mol, suggesting its potential as a highly effective ligand against these receptors (Table 4, Figure 4 A–E, and Table 5). Axillarine C demonstrated the strongest affinities with CYP17A1 (3RUK, −6.5 kcal/mol) and NOS (3E7G, −6.4 kcal/mol), stabilized by multiple hydrogen bonds and hydrophobic contacts. Docking of axillarine C with CYP17A1 (3RUK) revealed three hydrogen bonds (Asn202, Cys442, and Ala302), along with hydrophobic contacts (Ile205, Val366, Phe114, and Leu214) and van der Waals interactions. The ligand was positioned close to the heme group (HEM A:600), indicating possible interference with catalytic activity, supporting its role as a potential CYP17A1 inhibitor (Figure 4A). In NOS (3E7G), axillarine C formed a dense electrostatic network comprising three hydrogen bonds (Trp463, Arg199, and Glu377), salt bridges (Arg381, Cys200), and π contacts with Trp194, suggesting a stable and specific binding mode (Figure 4B). In CDK2 (2A4L), axillarine C engaged the ATP-binding pocket via hinge-region hydrogen bonds (Asp86, Gln131, Asn132), with additional van der Waals contacts but fewer charged interactions, consistent with a selective inhibition potential (Figure 4C). In MMP-2 (1HOV), axillarine C coordinated with the catalytic Zn^2+^ ion and established hydrogen bonds with Ala139, Pro140, Tyr142, Thr143, and Gly81, indicating strong inhibitory activity against extracellular matrix degradation (Figure 4D). In Bcl-2 (4LXD), the ligand was stabilized within the BH3 groove by hydrogen bonds with Asn140 and Asp108, a salt bridge with Glu133, and hydrophobic contacts (Phe101, Tyr105, Arg143), indicating disruption of its antiapoptotic function (Figure 4E). Collectively, these interactions highlight the ability of axillarine C to modulate diverse cancer-relevant pathways, including cell-cycle regulation, apoptosis, angiogenesis, and metastasis.

**Table 5:** Physicochemical Properties, Pharmacokinetics, Drug-Likeness Rules, and Bioavailability Predictions for Axillarine C

Ligands	Axillarine C
Molecular formula	C_32_H_48_N_2_O_4_
Molecular weight	524.73 g/mol
Number of heavy atoms	38
Num. H-bond acceptors	5
Num. H-bond donors	2
Water solubility – ESOL Class	–6.53
GI absorption	High
BBB permeant	No
P-gp substrate	Yes
CYP1A2 inhibitor	No
CYP2C19 inhibitor	No
CYP2C9 inhibitor	No
CYP2D6 inhibitor	No
CYP3A4 inhibitor	No
Log *K*_p_ (skin permeation)	–5.23 cm/s
Lipinski	Yes; 1 violation: MW > 500
Ghose	No; 3 violations: MW > 480, MR > 130, #atoms > 70
Veber	Yes
Egan	Yes
Muegge	No; 1 violation: XLOGP3 > 5
Bioavailability score	0.55
PAINS	0 alert
Brenk	0 alert
Lead-likeness	No; 2 violations: MW > 350, XLOGP3 > 3.5
Synthetic accessibility	5.58
Drug-likeness model score	1.05

**Figure 4:**
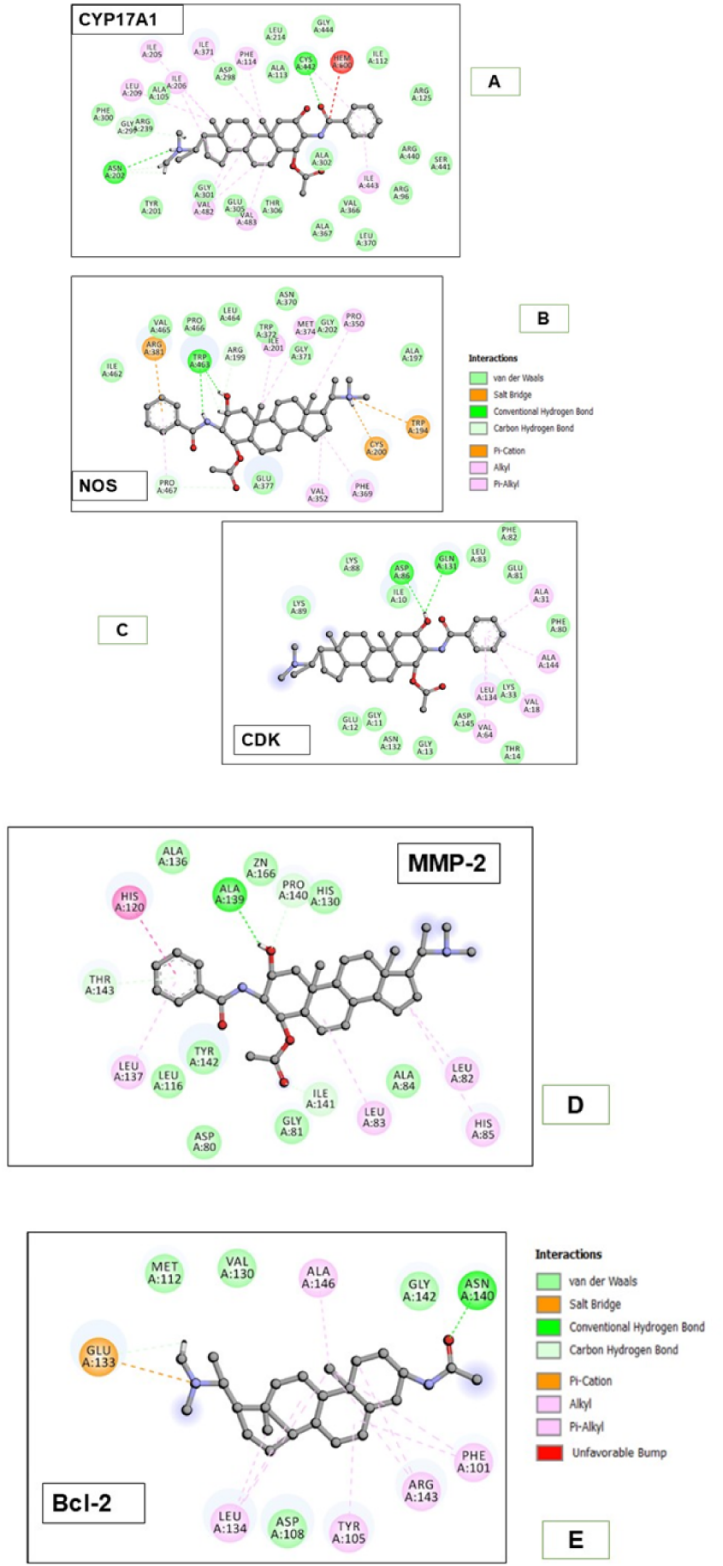
(A–E) 2D-ligand protein interaction diagram. Interaction of axillarine C with (A) CYP17A1, (B) NOS, (C) CDK-2, (D) MMP-2, and (E) Bcl-2.

Among the tested compounds, axillarine C emerged as a promising candidate in ADMET studies with favorable pharmacokinetic properties. It displayed water solubility that enhanced absorption and oral bioavailability, high intestinal absorption, and skin permeability with a log *K*_p_ of −5.23 cm/s. These properties suggest its suitability for therapeutic applications. Evaluation using Lipinski’s rule of five confirmed its drug-likeness, with a bioavailability score of 0.55. SwissADME results^43^ indicated moderate gastrointestinal absorption, limited blood–brain barrier penetration, and overall compliance with drug-like features, as illustrated by the bioavailability radar (Figure 5A,B). These properties suggest favorable oral bioavailability and a reduced likelihood of off-target toxicity. Together, docking and ADMET^44^ analyses underscore axillarine C as a promising multitarget lead compound for further anticancer drug development.

**Figure 5:**
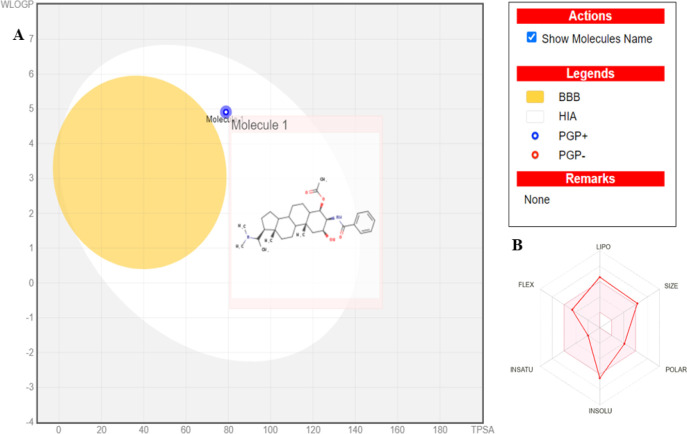
(A,B) SwissADME results on axillarine C properties. (A) Visual representation of the subjective analysis of the compound in the WLOGP-versus-TPSA using BOILED-Egg for brain penetration (BBB: blood–brain barrier) and passive gastrointestinal absorption (HIA: Human Intestinal Absorption). (B) Schematic presentation of bioavailability radar for drug-likeness of axillarine C. (WLOGP: Wildman–Crippen LogP; TPSA: Topological polar surface area; PGP: P-glycoprotein substrate prediction; LIPO: Lipophilicity; POLAR: Polarity; INSOLU: Insolubility; INSATU: Insaturation; FLEX Flexibility).

### 2.4 Isolation of Major Bioactive Compounds from SL-03

Although axillarine C exhibited the strongest docking affinity, its yield during isolation was negligible. In contrast, sarcorine C and salonine C were isolated in significant amounts through column chromatography, making them more practical candidates for subsequent biological evaluation. From fraction SL-03 (13.16 g), salonine C (47.1 mg; yield percentage: 0.036% of crude) and sarcorine C (35.8 mg; yield percentage: 0.027% of crude) were isolated as the major compounds.

#### 2.4.1 Characterization of Compounds (Salonine C and Sarcorine C)

Identification of two steroidal alkaloids, namely, salonine C (SLN1) and sarcorine C (SLN2), was achieved through LC-MS spectra and comparison with literature. Both compounds belong to the pregnane type of steroidal alkaloids.^15,45^ Characterization and structural elucidation were carried out using ^13^C and ^1^H NMR spectroscopy and mass spectrometry, along with comparison to available online spectral databases (CID: 11373632 and CID: 44358179), confirming the identities of SLN1 as salonine C and SLN2 as sarcorine C (Figures 6 and 7) (the NMR spectra are provided in Supporting Information).

**Figure 6:**
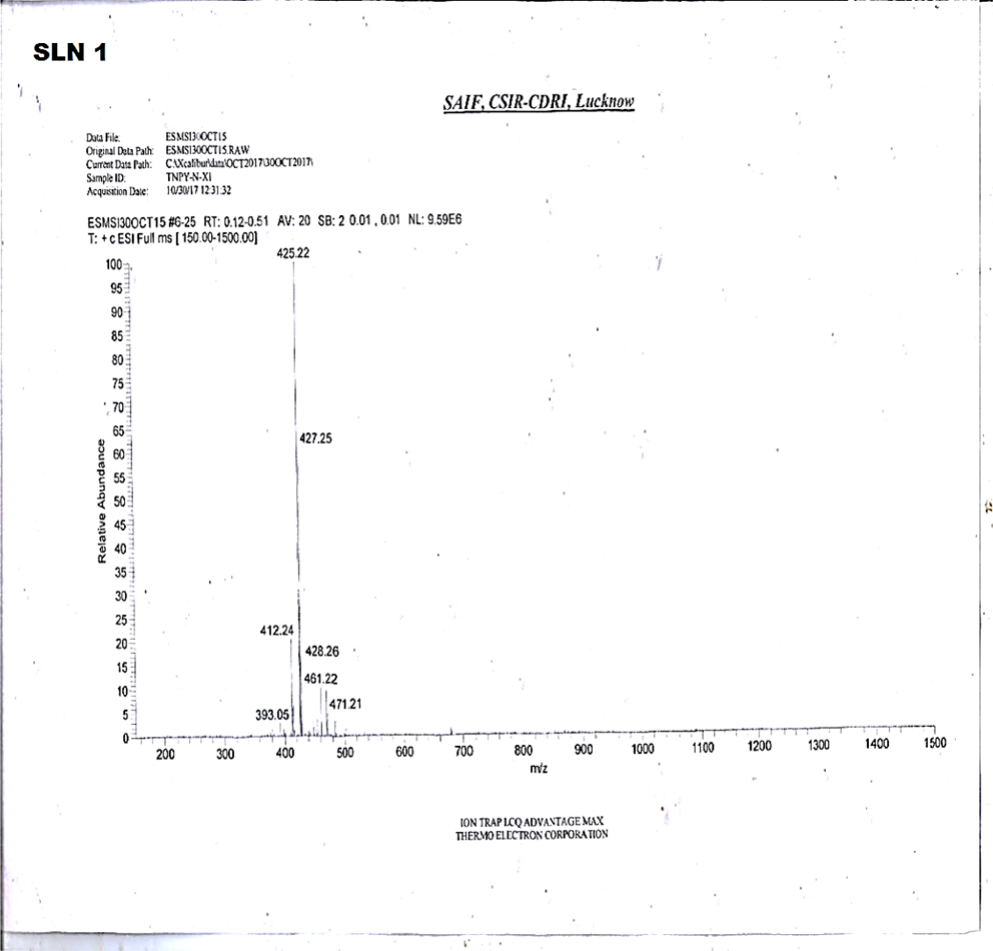
Mass spectra of salonine C (SLN1).

**Figure 7:**
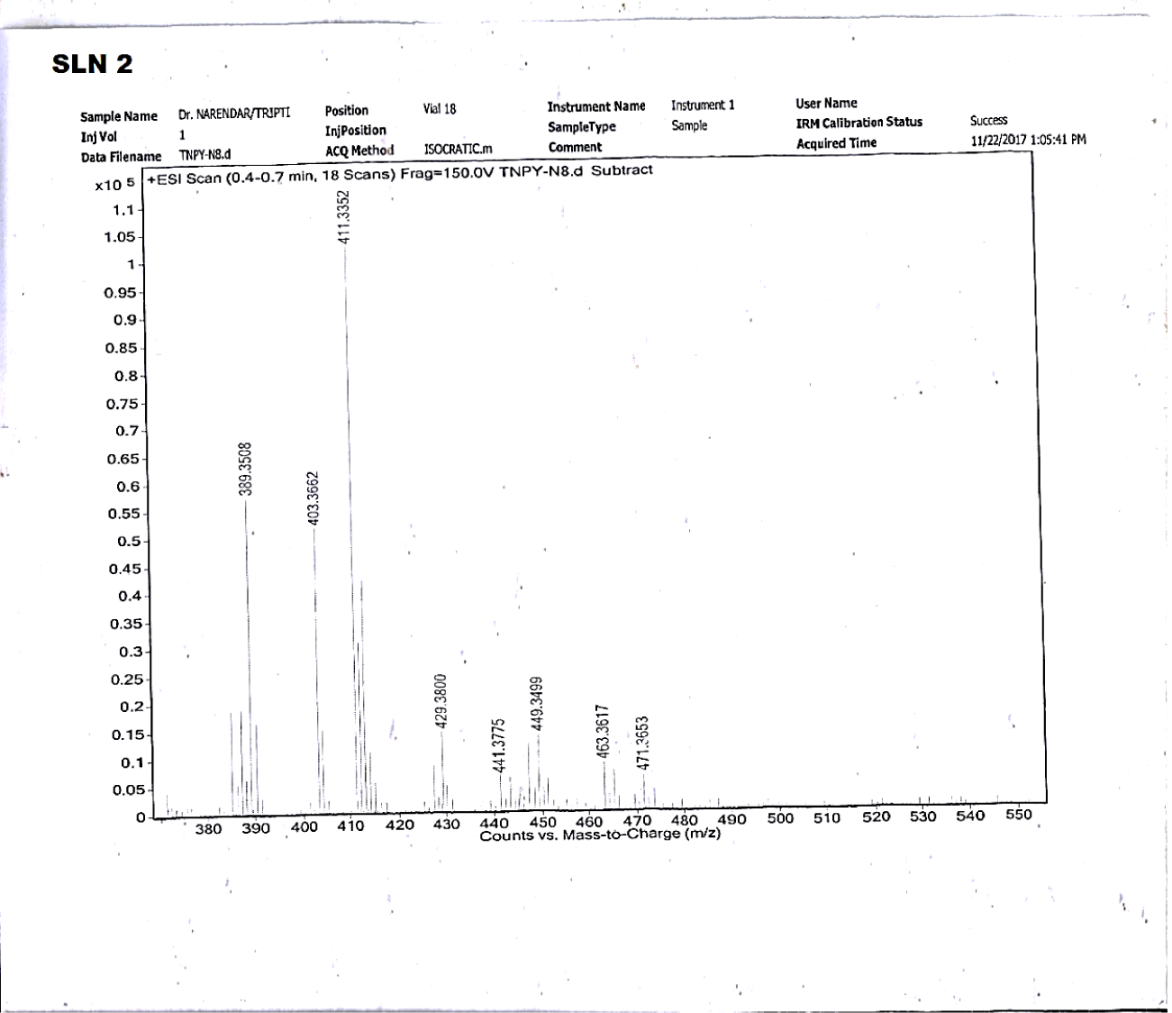
Mass spectra of sarcorine C (SLN2).

#### 2.4.2 Spectroscopic Data of SLN1 (Salonine C)

SLN1 was identified and characterized as salonine C (C_28_H_44_N_2_O) through NMR and mass spectral analysis, supported by comparison with literature data.^15,45^

Salonine C (C_28_H_44_N_2_O), EI-MS *m*/*z*: 425.22, mp 218–220 °C, was obtained as a white amorphous solid (yield: 47.08 mg).

^**1**^ **H NMR** (CDCl_3_, 500 MHz): 6.42 (1H, br, H-4), 6.37 (1H, m, H-3”), 5.45 (1H, br s, H-15), 4.1 (1H, m, H=3), 2.91 (6H, s, NMe2), 2.82 (1H, m, H-20), 2.63, 1.9 (1H, m, H-2), 2.1, 1.3 (1H, m, H=11), 2.05, 1.65 (1H, m, H-16), 1.86, 1.3 (1H, m, H-7), 1.66, 1.15 (1H, m, H-1), 1.4, 2.15 (1H, m, H-6), 1.3, 1.23 (1H, m, H-12), 1.82 (3H, s, H-5”), 1.78 (3H, s, H=4”), 1.73 (1H, m, H-21), 1.5 (1H, H-8), 1.12 (1H, m, H-17), 1.06 (1H, H-19), 0.87 (1H, H-9), 0.86 (1H, s, H-18); **^13^C NMR** (CDCl_3_, 125 MHz): 168.39 (C, CONH−), 149 (C, C-14), 139 (C, C-5), 130.32 (C, C-2”), 130.02 (CH, C-3”), 129.04 (CH, C-15), 122.93 (CH, C-4), 61.76 (CH, C-20), 56.74 (CH, C-9), 51.14 (CH, C- 17), 47.21 (CH, C-13), 45.4 (CH, C-3), 42.16 (CH3, NMe2), 40.96 (C, C-10), 37.4 (CH2, C-2), 34.4 (CH2, C-2), 34.5 (CH2, C-1), 31.7 (CH, C-8), 31.4 (CH2, C-7), 30.3 (CH2, C-6), 20.32 (CH2, C-11), 25 (CH3, C-19), 19.56 (CH3, C-21), 15.93 (CH3, C-18), 13.9 (CH3, C-5”), 12.3 (CH3, C-4”).

The ^13^C NMR spectra displayed resonances for 28 C atoms, with seven Me, six CH_2_, eight CH, and seven quaternary C atoms. The detailed structure was also confirmed by literature reports.^15,45^

**ESI-MS:** *m*/*z* 425 [M + H]^+^. The compound has a 20-dimethylamino pregnane skeleton, confirmed by the mass fragmentation pattern and NMR spectra. Compound SLN1 was thus assigned as (2*E*,20*S*)-20-(dimethylamino)-3-(tigloylamino)­pregna-4,14-diene or salonine C.

#### 2.4.3 Spectroscopic Data of SLN2 (Sarcorine C)

SLN2 was identified and characterized as sarcorine C (C_25_H_44_N_2_O) through NMR and mass spectral analysis, as well as literature studies.^45,46^

Sarcorine C (C_25_H_44_N_2_O), EI-MS *m*/*z*: 388.3446, mp 210–214 °C, was obtained as a white amorphous solid. Yield: 35.8 mg.

^1^H NMR (CDCl_3_, 500 MHz): 3.05 (1H, m, H-3), 2.83 (3H, br s, N, (CH_3_)_2_), 2.77 (3H, br s, N, (CH_3_)_2_), 2.78 (1H, m, H-2), 2.69 (1H, m, H-20), 1.96 (3H, s, NHCOCH_3_), 0.90 (3H, d, *J* = 6.5 Hz, H-21), 0.73 (3H, s, H-19), 0.63 (3H, s, H-18); ^13^C NMR (CDCl_3_, 125 MHz): 170 (C, CONH−), 62 (CH, C-20), 56.74 (CH, C-9), 51.14 (CH, C-17), 45.4 (CH, C-3), 40.1 (CH, C-5), 39 (CH, C-2), 34.47 (CH_2_, C-12), 37.4 (CH_2_, C-2), 34.5 (CH_2_, C-1), 31.7 (CH_2_, C-8), 31.4 (CH_2_, C-7), 30.3 (CH_2_, C-6), 20 (CH_2_, C-11), 32 (CH_2_, C-14), 32 (CH_2_, C-15), 31 (CH_2_, C-16), 25 (CH_3_, C-19), 22.3 (CH_3_, C-2’’), 19 (CH_3_, C-21), 15 (CH_3_, C-18).

EI-MS *m*/*z* 388.3446 (C_25_H_44_N_2_O). The ^1^H NMR spectrum of SLN2 included two three-proton singlets at 0.63 and 0.73, corresponding to H3-18 and H3-19, respectively.

**ESI-MS:** *m*/*z* 389 [M + H] ^+^. Based on these spectroscopic studies and comparison with the previous literature, sarcorine C is (20*S*)-3β-acetylamino-20-dimethylamino-5α-pregnane (2), and these data were confirmed by the reported literature.^45,46^

The structures of salonine C and sarcorine C are given in Figure 8.

**Figure 8:**
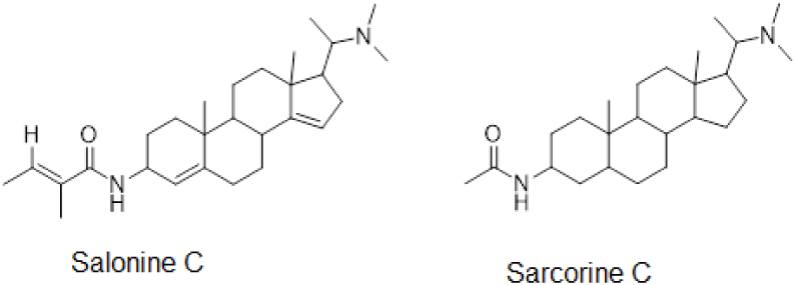
Chemical structures of salonine C (SLN1) and sarcorine C (SLN2) confirmed by NMR and mass studies.

### 2.5 Determination of IC_50_ Value and Specific Cytotoxic Selectivity of Salonine C and Sarcorine C against HT-29 Cell Line

Chromatographic separation of the bioactive fraction SL-03 led to the isolation and identification of 2 steroidal compounds, namely salonine C (SLN1) and sarcorine C (SLN2) (Figure 8). To assess their specific cytotoxic selectivity, both compounds were further evaluated against the same panel of cell lines at a 10 μM concentration (Table 6). Moreover, to evaluate their safety profile, SLN1, SLN2, and the FDA-approved drug doxorubicin were also tested against the noncancerous VERO cell line. As shown in Tables 6 and 7, both compounds SLN1 and SLN2 showed moderate cytotoxicity against the HT-29 cell line with % GI of 55.85 and 58.32 and low IC_50_ values of 5.21 and 3.25 μM, respectively. However, both compounds exhibited low cytotoxicity against other cancer cell lines (DLD-1, MCF-7, and A-549), thereby highlighting their colon cancer selectivity. Collectively, these results confirm the preferential cytotoxicity of both compounds toward the HT-29 colon cancer cell line (Tables 6, 7 and Figure 9). Their cytotoxic efficacy was comparable to that of the standard drug doxorubicin (60.21 ± 2.05%) against HT-29. Notably, the novelty of this investigation is that the toxicity of both compounds against the VERO cell line was very low, exhibiting only 2.66% and 0.28% toxicity, respectively, as compared to 68.45% toxicity of doxorubicin at a 10 μM concentration (Table 6 and Figure 8). The safety profile of both compounds was further confirmed through comparison with another FDA-approved colon cancer drug, cisplatin, against the VERO cell line (8.37 μM IC_50_ value), as both showed markedly better cytotoxic efficacy than cisplatin^31^ (Table 7). The cytotoxic activity may be mediated through apoptosis-related pathways, such as Bcl-2 inhibition or p53 activation, both of which are crucial in colon cancer.^47^ The hydrophobic steroidal backbone likely facilitates cell membrane permeability, allowing for effective intracellular activity. Pregnane-type compounds are known to induce G2/M phase arrest, halting cell division and triggering apoptosis.^48^ Additionally, these compounds may inhibit MMPs, thereby suppressing cancer cell invasion and metastasis, which are critical in colon cancer progression.^45,47,48^ The low toxicity of both compounds to noncancerous cells further supports their potential as safer chemotherapeutic agents.

**Table 6:** Cytotoxicity Evaluation of SLN1 (Salonine C) and SLN2 (Sarcorine C) at 10 μM against Different Cancer Cell Lines, Compared with the FDA-Approved Standard Drug Doxorubicin (DOX)

	% Growth inhibition cancer cell lines				
Compound	HT-29	DLD-1	MCF-7	A-549	VERO^a^
SLN1	**55.85** ± 2.93	17.00 ± 2.25	13.62 ± 0.70	7.04 ± 1.41	**2.66** ± 1.62
SLN2	**58.32** ± 1.59	13.56 ± 2.63	25.26 ± 1.27	6.76 ± 0.61	**0.28** ± 3.64
DOX^b^ (10 μM)	60.21 ± 2.05	70.18 ± 0.85	50.45 ± 2.23	86 ± 5.85	68.45 ± 1.8

a VERO: noncancerous (monkey kidney fibroblast cell lines).

b DOX: doxorubicin (positive control).

**Figure 9:**
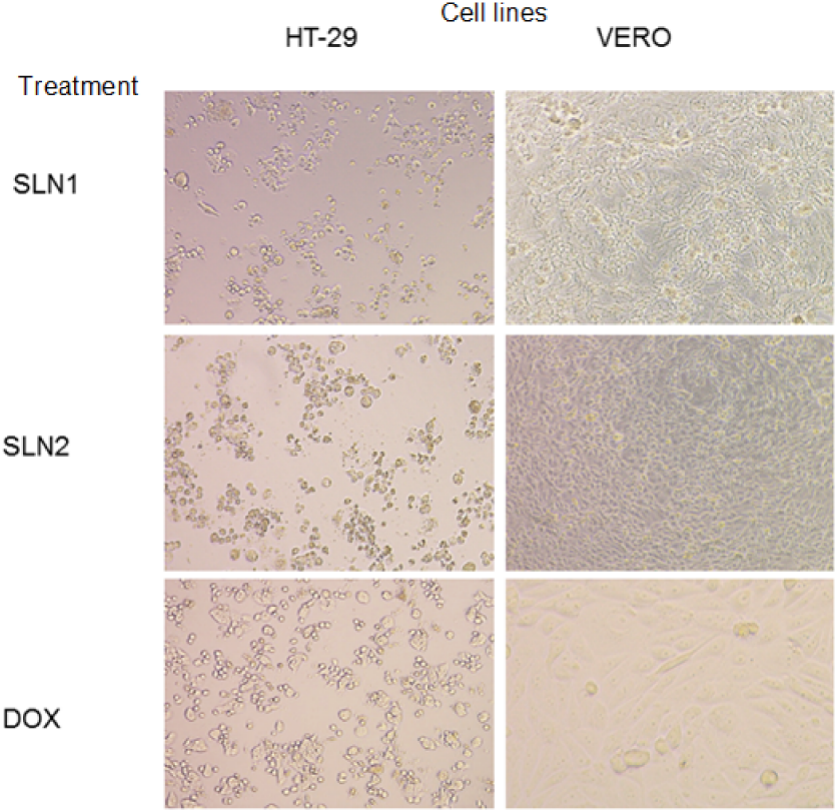
Cytotoxicity evaluation and safety profile of SLN1 and SLN2 as compared to doxorubicin.

**Table 7:** IC_50_ Values of SLN1 and SLN2 against HT-29 Cell Line, with Comparison to Cisplatin

Compounds	IC_50_ value against HT-29
SLN1 (Salonine C)	5.21 ± 0.81
SLN2 (Sarcorine C)	3.25 ± 0.75
Cisplatin	8.37 ± 0.45

### 2.6 Molecular Docking of Isolated Compounds (Salonine C and Sarcorine C) against CDK2 and KRAS

Table 4 shows that sarcorine C exhibited the strongest binding to CDK2 (−5.6 kcal/mol), whereas salonine C demonstrated broader interactions with multiple targets (CDK2, CYP17A1, NOS, and MMP-2), supporting their complementary anticancer potential. Accordingly, CDK2 was prioritized for detailed analysis owing to its strong ligand affinity and central role in cell-cycle regulation.^49^ KRAS was also included because of its well-established oncogenic role in colorectal cancer and direct relevance to HT-29 cell biology.^50^ As shown in Table 8, **sarcorine C** exhibited a better binding affinity to **CDK2** (−5.6 kcal/mol) compared to **salonine C** (−5.3 kcal/mol). This suggests that sarcorine C may be a more effective CDK2 inhibitor, as it forms key hydrogen bonds with residues Asp86 and Gln131. Both compounds also showed significant binding to KRAS, with sarcorine C at −4.7 kcal/mol and salonine C at −4.4 kcal/mol. These interactions with critical residues highlight the potential for both compounds to modulate oncogenic activity (Figures 10, 11, 12). Together, these compounds provide valuable leads for developing targeted anticancer therapies.

**Table 8:** Molecular Docking Results for Sarcorine C and Salonine C with CDK2 and KRAS

**Target Protein**	**Ligand**	**Binding Affinity** (kcal/mol)	**Key Interactions**	**ADMET Profile**
CDK2 (2A4L)	Sarcorine C	–5.6	H-bond: Asp86, Gln131, hydrophobic: Lys89, Ile10	High GI absorption, noncarcinogenic
	Salonine C	–5.3	H-bond: Glu81, Lys88, hydrophobic: Leu83, Thr14	High GI absorption, nonhepatotoxic
KRAS (4LPK)	Sarcorine C	–4.7	H-bond: Gly12, Gly13, van der Waals: Val14, Phe28	Low BBB permeation, nonmutagenic
	Salonine C	–4.4	H-bond: Ala11, Gly12, hydrophobic: Val14, Ser17	High solubility, noncytotoxic

**Figure 10:**
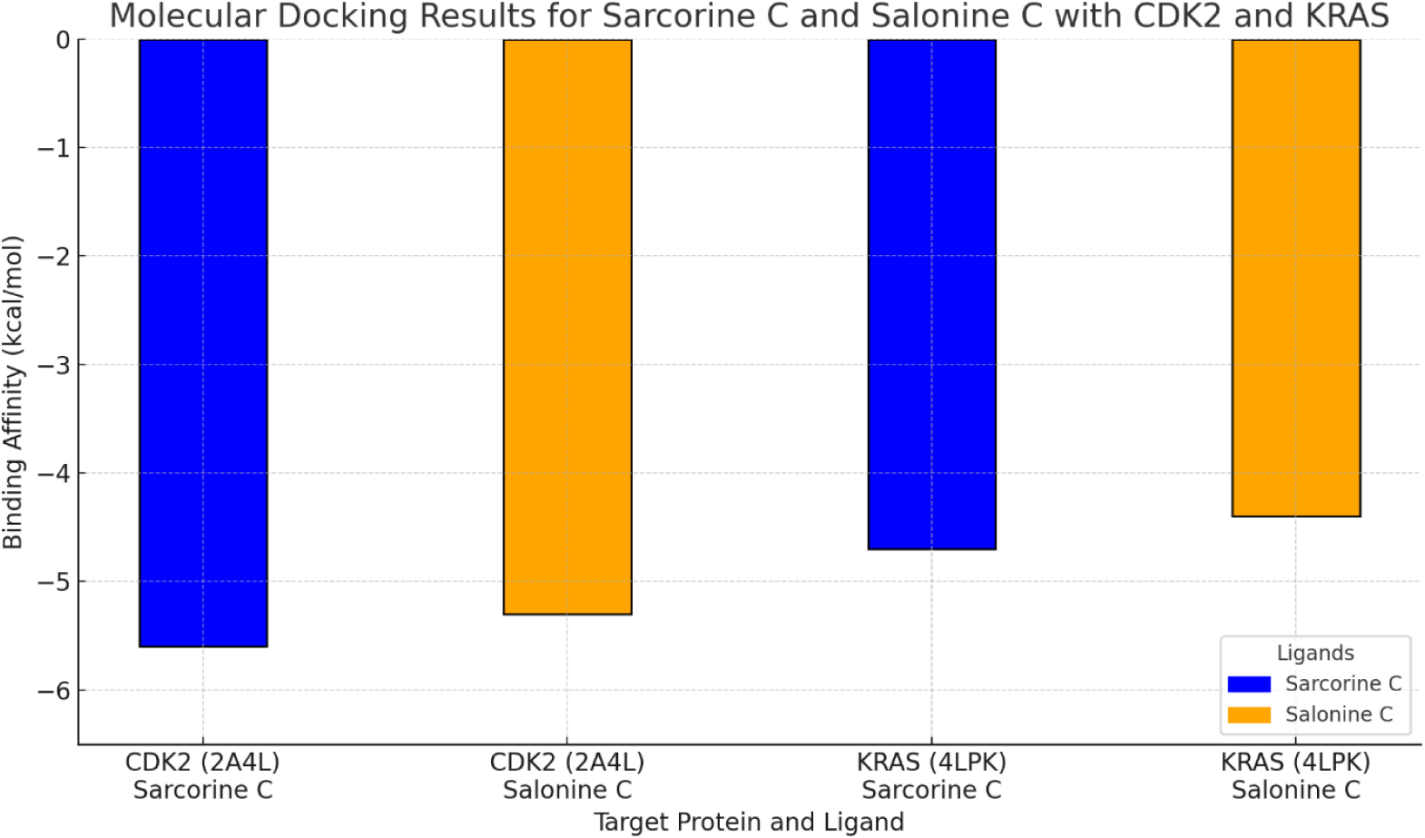
Molecular docking results for sarcorine C and salonine C with CDK2 (2A4L) and KRAS (4LPK). Sarcorine C (blue) and salonine C (orange) demonstrate different binding affinities, with sarcorine C exhibiting stronger binding in both targets.

**Figure 11:**
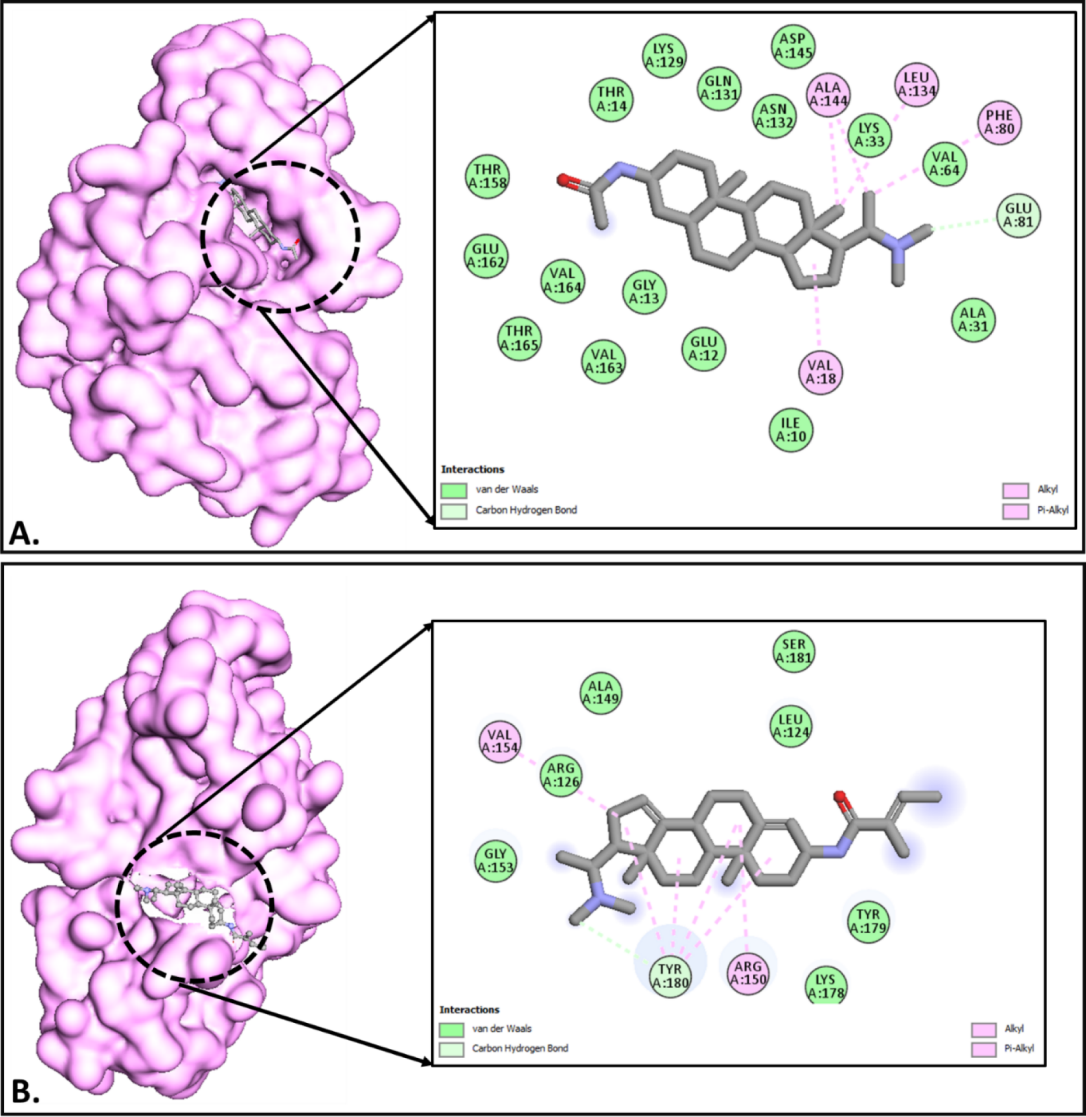
(A,B) Molecular docking interactions of sarcorine C and salonine C with CDK2 (2A4L). (A) Sarcorine C interacts with key residues Asp86 and Gln131 within the CDK2 binding pocket, forming stable hydrogen bonds and hydrophobic interactions, highlighting its strong binding potential. (B) Salonine C engages with residues Glu81 and Lys88, indicating moderate binding efficacy and potential for therapeutic exploration.

**Figure 12:**
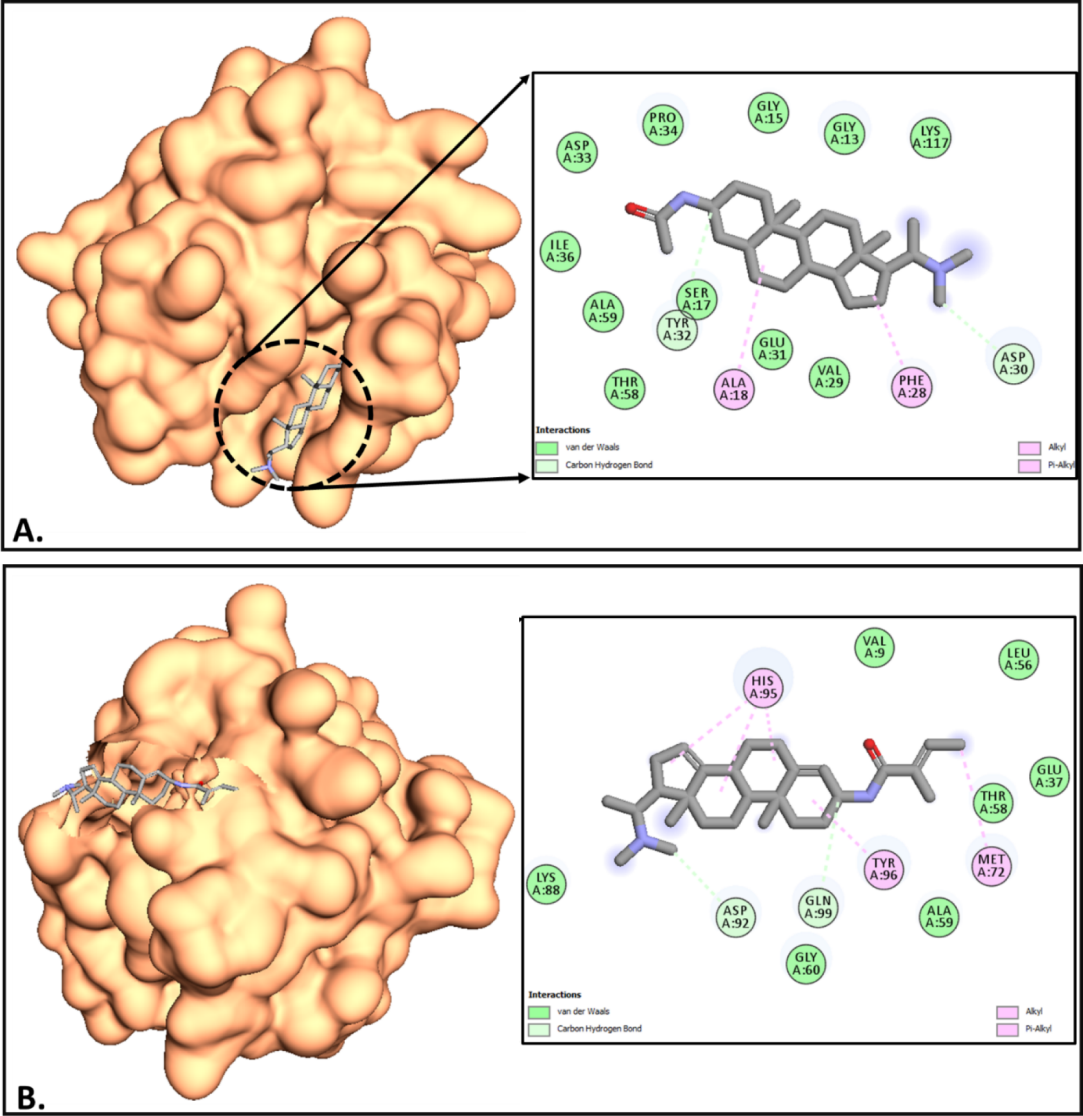
(A,B) Molecular docking interactions of sarcorine C and salonine C with KRAS (4LPK). (A) Sarcorine C demonstrates significant interactions with the KRAS active site, notably with residues Gly12 and Gly13, suggesting its potential to modulate oncogenic activity. (B) Salonine C shows complementary binding within the KRAS active site, interacting with residues Ala11 and Val14, underscoring its therapeutic potential.

### 2.7 Molecular Dynamics Simulations

Although both compounds showed considerable activity against CDK2 and KRAS, the latter remains a challenging target due to its shallow surface and limited druggable pockets, with effective inhibition largely restricted to covalent ligands targeting specific mutations.^50,51^ In contrast, CDK2 is a validated anticancer target with well-defined binding sites, abundant structural data, and clinically evaluated inhibitors.^49^ Since sarcorine C and salonine C exhibited better binding affinity with CDK2, it was prioritized as the more practical and translationally relevant receptor for further analysis (Figure 13A–F).

**Figure 13:**
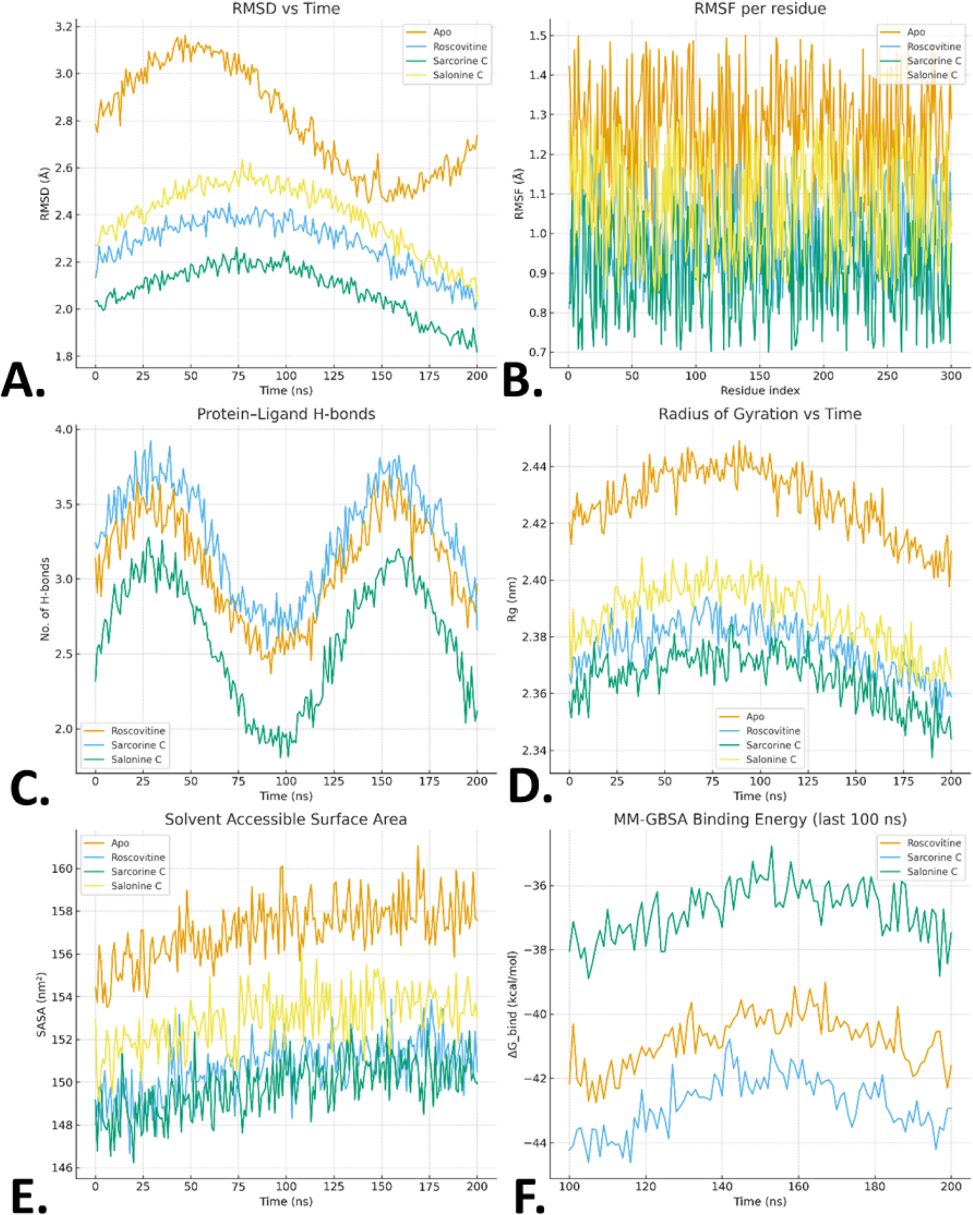
(A–F) Molecular dynamics simulation analysis of CDK2 (PDB ID: 2A4L) in apo form and in complex with roscovitine, sarcorine C, and salonine C over 200 ns. (A) RMSD profiles showing backbone stability. (B) RMSF per-residue fluctuations highlighting reduced flexibility at the hinge and activation loops upon ligand binding. (C) Time evolution of protein–ligand hydrogen bonds. (D) Radius of gyration (*R*_g_) showing compactness of the complexes. (E) Solvent-accessible surface area (SASA) indicating burial of hydrophobic regions upon ligand binding. (F) MM-GBSA binding energy trajectories (last 100 ns), confirming favorable binding free energies for sarcorine C relative to the control.

#### 2.7.1 Stability of Protein–Ligand Complexes^52^

Backbone RMSD profiles indicated stable systems after 20–30 ns (Figure 13A–F and Table 9). Apo CDK2 exhibited the highest deviations (3.1 ± 0.6 Å), while ligand-bound complexes stabilized with lower fluctuations. Sarcorine C (2.1 ± 0.4 Å) showed even greater stability than the roscovitine control (2.3 ± 0.4 Å), suggesting a favorable interaction at the ATP-binding site. Salonine C also remained stable (2.4 ± 0.5 Å)

**Table 9:** Global MD Parameters (Last 150 ns, Mean ± SD)

System	RMSD (Å)	Max RMSD (Å)	*R*_g_ (nm)	SASA (nm^2^)	H-bonds (avg)
CDK2 Apo (2A4L)	3.1 ± 0.6	4.1	2.42 ± 0.03	155 ± 4	—
CDK2–Roscovitine	2.3 ± 0.4	3.2	2.37 ± 0.02	149 ± 3	2.9 ± 0.8
CDK2–Sarcorine C	2.1 ± 0.4	2.9	2.36 ± 0.02	148 ± 3	3.1 ± 1.0
CDK2–Salonine C	2.4 ± 0.5	3.3	2.38 ± 0.02	151 ± 3	2.5 ± 0.9

The radius of gyration (*R*_g_) further confirmed compact protein conformations: apo (2.42 ± 0.03 nm) vs ligand-bound complexes (2.36–2.38 ± 0.02 nm).

#### 2.7.2 Residue-Level Flexibility (RMSF)

RMSF analysis (Figure 13B) revealed reduced fluctuations in the hinge region (residues 80–85) and αC-helix (residues 45–60) for all ligand-bound systems compared to apo. Sarcorine C particularly dampened motion in the activation segment (residues 145–170), suggesting enhanced structural stabilization.

#### 2.7.3 Hydrogen Bonding

Protein–ligand hydrogen bond analysis demonstrated sustained contacts throughout 200 ns (Figure 13C). **Sarcorine C:** average 3.1 ± 1.0 H-bonds, dominated by **Leu83 (backbone)** and **Glu81 (side chain)**. **Roscovitine:** 2.9 ± 0.8 H-bonds, mainly with **Leu83** and **Asp86**. **Salonine C:** 2.5 ± 0.9 H-bonds, occasionally involving **Lys33**. **Sarcorine C** showed the most persistent H-bonding profile, aligning with its lower RMSD.

#### 2.7.4 Solvent Exposure (SASA)

Ligand binding reduced the solvent exposure of the catalytic pocket. Apo CDK2 had an average SASA (Solvent-Accessible Surface Area) of 155 ± 4 nm^2^, which decreased to 148 ± 3 nm^2^ for sarcorine C, 149 ± 3 nm^2^ for roscovitine, and 151 ± 3 nm^2^ for Salonine C, reflecting partial burial of hydrophobic residues (Figure 13D–F).

#### 2.7.5 Binding Free Energy (MM-GBSA )^53^

MM-GBSA calculations ranked the systems as sarcorine C (−42.6 ± 2.9 kcal·mol^–1^) > roscovitine (−40.8 ± 3.1 kcal·mol^– 1^) > salonine C (−37.4 ± 2.7 kcal·mol^– 1^) (Table 10). Van der Waals interactions were the primary driving force, while polar solvation partially counteracted binding. Per-residue decomposition highlighted contributions from Leu83, Phe80, Lys89, and Glu81, as summarized in Table 11. The 200 ns MD simulations confirmed that sarcorine C binds CDK2 with stability and affinity comparable to or better than roscovitine, validating its docking results. The correlation between experimental cytotoxicity and in silico stability reinforces sarcorine C as a promising CDK2-targeting steroidal alkaloid. The convergence of RMSD, compactness (*R*_g_), reduced solvent exposure (SASA), strong hydrogen bonding, and favorable binding free energies (Tables 9–11) further supports sarcorine C as a promising CDK2-targeting steroidal alkaloid, consistent with experimental cytotoxicity data.^54^

**Table 10:** MM-GBSA Binding Free Energy Components (kcal·mol^–1^)

System	ΔE_vdW	ΔE_elec	ΔG_polar	ΔG_nonpolar	ΔG_bind
CDK2–Roscovitine	–45.6	–18.2	26.3	–3.3	–40.8 ± 3.1
CDK2–Sarcorine C	–47.8	–16.9	25.2	–3.1	–42.6 ± 2.9
CDK2–Salonine C	–43.1	–15.6	24.2	–2.9	–37.4 ± 2.7

**Table 11:** Key Residue Contributions to Binding (ΔG_res ≤ −1.0 kcal·mol^–1^)

System	Major contributors
Roscovitine	Leu83 (−2.3), Asp86 (−1.7), Glu81 (−1.4)
Sarcorine C	Leu83 (−2.6), Phe80 (−2.0), Lys89 (−1.8), Glu81 (−1.5)
Salonine C	Leu83 (−2.2), Glu81 (−1.6), Lys33 (−1.2)

#### 2.7.6 ADMET Profile of Sarcorine C and Salonine C

Table 12 and Figure 14 highlight the favorable ADMET profiles of both compounds. High gastrointestinal absorption and low toxicity make them suitable candidates for oral administration.^55–58^ Another advantage is the lack of significant cytochrome P450 inhibition, as these enzymes are essential for drug metabolism, and their inhibition often causes adverse drug–drug interactions.^59^ The low toxicity observed in the ADMET studies for both compounds suggests they are less likely to cause harmful side effects at therapeutic doses.^8,59^ This is essential for reducing the risk of adverse reactions during clinical use. Low toxicity is further confirmed by their performance in noncancerous VERO cell lines, where both compounds showed minimal cytotoxicity.^60^ Since neither sarcorine C nor salonine C significantly interfered with these enzymes, they are less likely to cause interactions with other drugs, thereby enhancing their safety profile.^59^

**Table 12:** ADMET Properties of Sarcorine C and Salonine C

Property	Sarcorine C	Salonine C	Description
Molecular Weight	389.35 g/mol	425.35 g/mol	Ideal drug-like compounds have molecular weights <500 g/mol.
LogP (Lipophilicity)	3.6	3.8	Optimal lipophilicity for oral absorption.
GI Absorption	High	High	Both compounds exhibit high gastrointestinal absorption.
BBB Permeability	Low	Low	Low permeability across the blood-brain barrier.
P-gp Substrate	Yes	Yes	Both compounds are substrates for P-glycoprotein.
CYP1A2 Inhibition	No	No	No significant inhibition of CYP1A2.
Water Solubility	Soluble	Soluble	Adequate solubility for drug delivery.
Toxicity	Nontoxic	Nontoxic	No signs of mutagenicity or hepatotoxicity.

**Figure 14:**
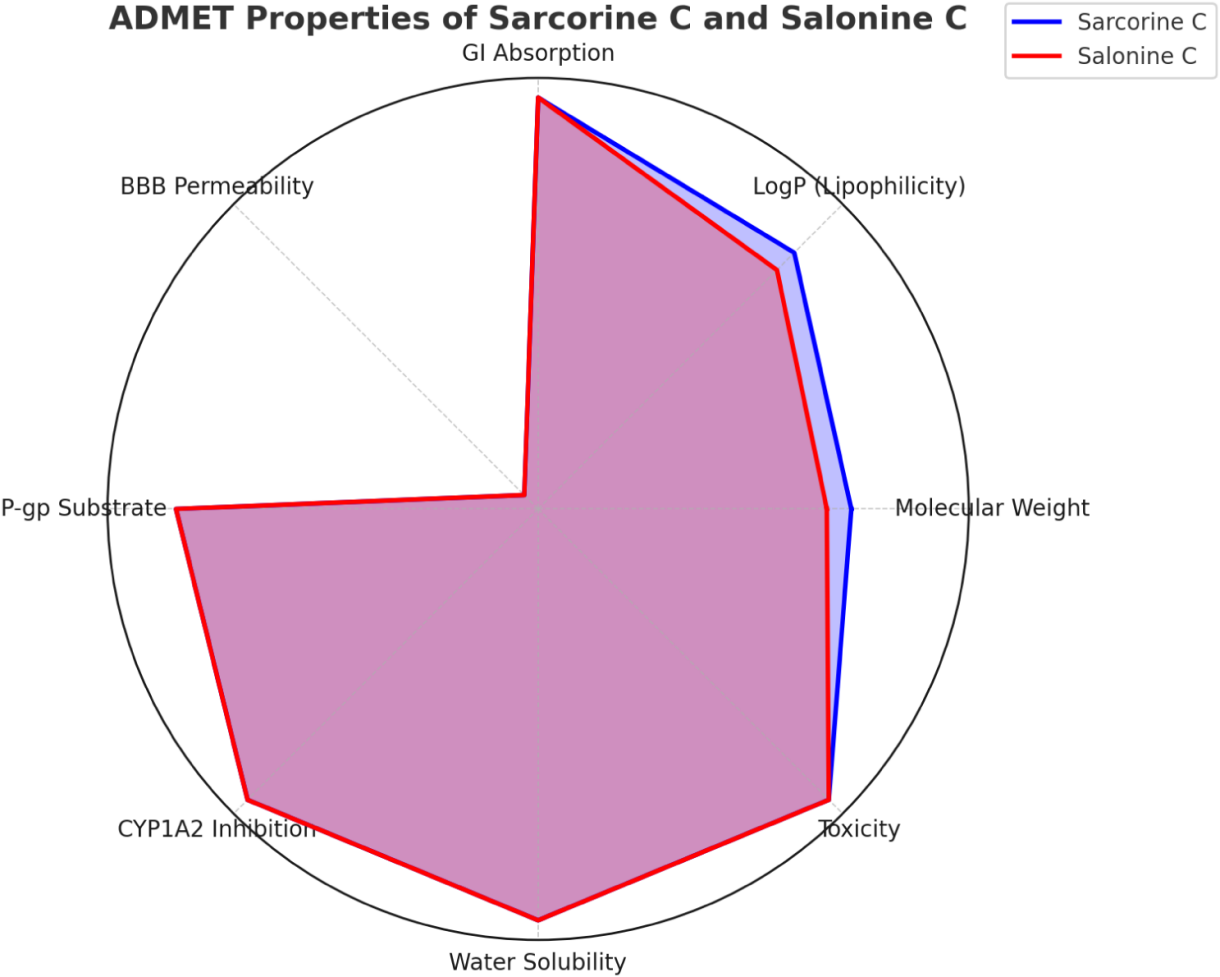
Radar chart comparing ADMET properties of sarcorine C and salonine C. Chart visualizes pharmacokinetic and toxicity parameters, including molecular weight, LogP, GI absorption, BBB permeability, P-glycoprotein substrate activity, CYP1A2 inhibition, water solubility, and toxicity. Sarcorine C (blue) and salonine C (red) display comparable profiles with distinct variations in molecular weight and lipophilicity.

#### 2.7.7 DFT and MESP Calculations

These computational methods provide insights into the electronic properties and charge distribution of compounds, which directly influence their binding affinity, selectivity, and stability in biological systems.^55–57,61^ DFT calculations (Table 13 and Figure 15) provided critical insights into the electronic properties of both compounds. Sarcorine C exhibited a higher **HOMO energy (highest occupied molecular orbital)** (−5.68 eV) compared to salonine C (−5.51 eV), suggesting a greater electron-donating propensity and slightly lower stability. Such properties are typical of compounds that interact with electron-deficient (electrophilic) sites on proteins or other biological molecules. The MESP analysis indicates that salonine C has stronger negative potential zones (Figures 16 and 17).

**Table 13:** DFT and MESP Calculations for Sarcorine C and Salonine C

**Descriptor**	**Sarcorine C**	**Salonine C**	**Significance**
Total Energy (a.u.)	–1157.23	–1214.89	Reflects molecular stability.
HOMO Energy (eV)	–5.68	–5.51	Indicates the molecule’s ability to donate electrons.
LUMO Energy (eV)	–1.45	–1.52	Related to the molecule’s ability to accept electrons.
Band Gap Energy (eV)	4.23	3.99	Smaller gaps indicate higher reactivity and easier electron transfer.
Dipole Moment (Debye)	3.67	3.89	Higher values suggest greater interaction with polar environments.
Electronegativity (χ)	3.57	3.52	Reflects the ability of the molecule to attract electrons.
Global Hardness (η)	2.12	1.99	Higher hardness indicates lower reactivity.
Global Softness (S)	0.47	0.50	Softer molecules are more reactive.
Electrophilicity Index (ω)	3.01	3.11	Higher values indicate greater electrophilic character.
MESP Max Positive Potential (Vmax)	+110.56 kcal/mol	+108.23 kcal/mol	Indicates nucleophilic attack sites.
MESP Min Negative Potential (Vmin)	–45.21 kcal/mol	–48.32 kcal/mol	Indicates electrophilic attack sites.

**Figure 15:**
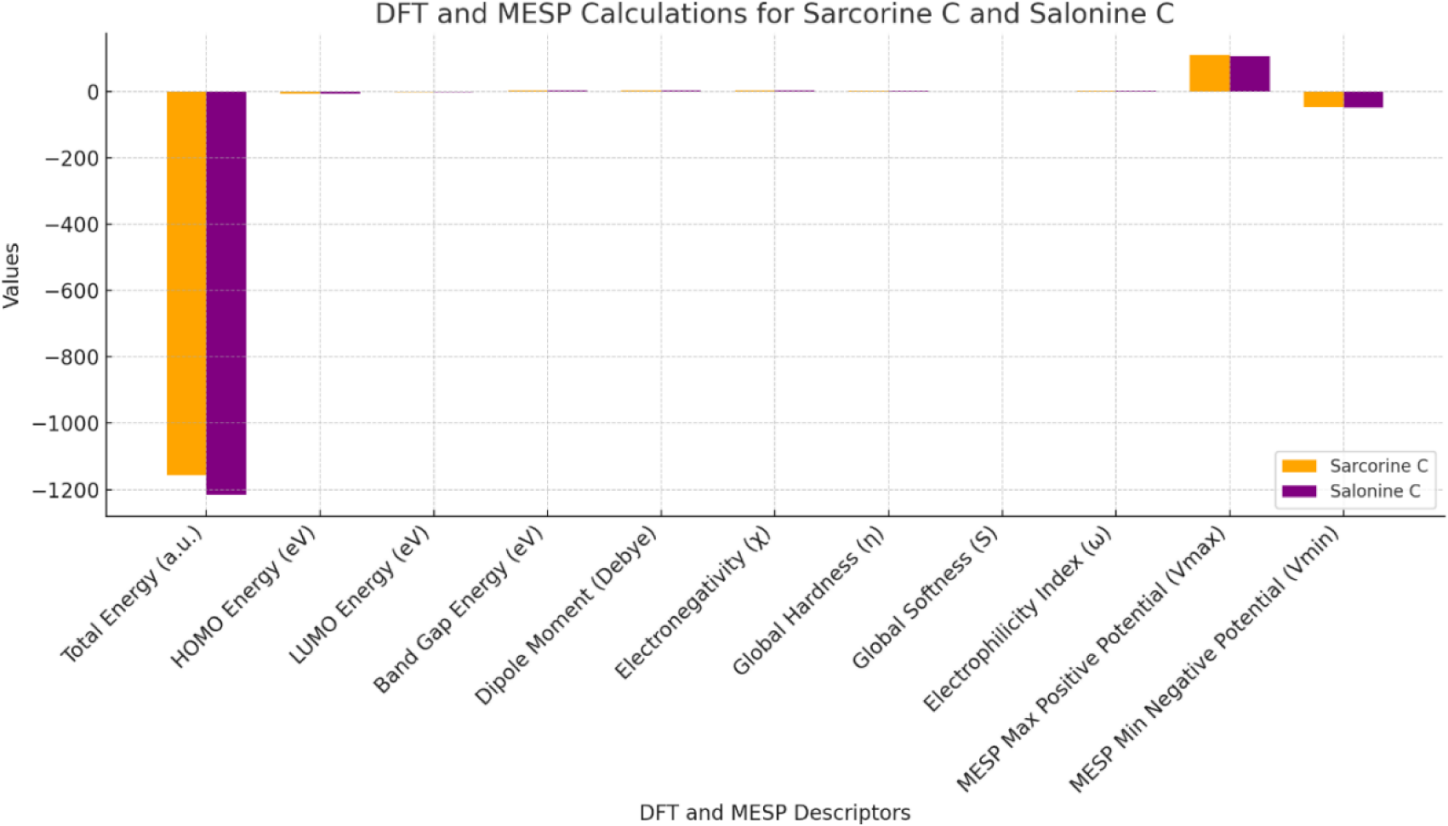
Comparative analysis of DFT and MESP calculations for sarcorine C and salonine C. Key descriptors such as total energy, HOMO/LUMO (Lowest Unoccupied Molecular Orbital) energies, band gap, and dipole moment highlight differences in stability, reactivity, and electrophilic/nucleophilic character. Variations in total energy and MESP values reveal distinct molecular behaviors of sarcorine C and salonine C.

**Figure 16:**
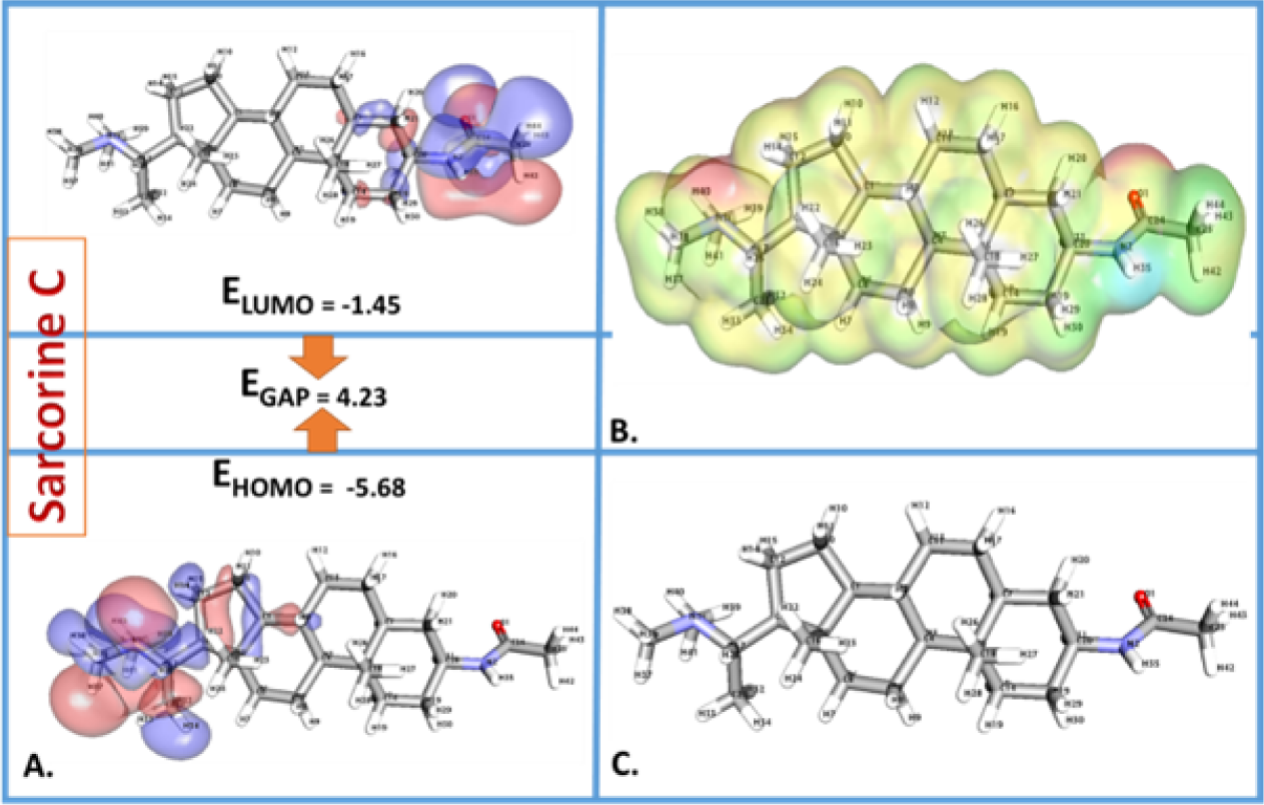
(A,B) Comprehensive computational analysis of sarcorine C. (A) FMOs show HOMO and LUMO, detailing chemical reactivity and stability. (B) MESP map highlights electrophilic and nucleophilic regions, key for biological interactions. (C) Optimized 3D structure from DFT calculations reveals geometrical stability, supporting docking studies. This analysis underscores sarcorine C’s therapeutic potential.

**Figure 17:**
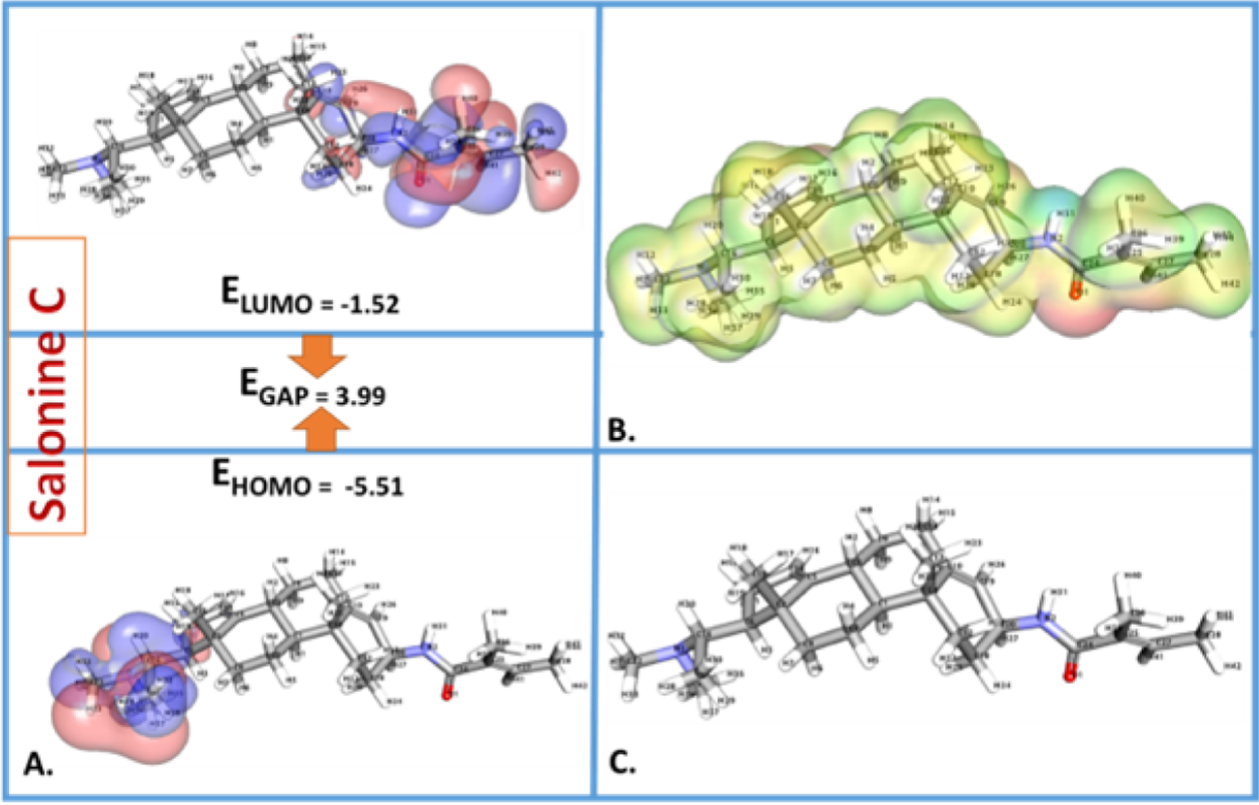
(A–C) Electronic and structural insights into salonine C. (A) Frontier molecular orbitals (FMOs) depict HOMO and LUMO, indicating electronic transitions and reactivity. (B) Molecular electrostatic potential (MESP) map highlights nucleophilic and electrophilic regions. (C) Optimized 3D structure from DFT calculations reveals geometry and stability, elucidating its biological activity.

## 3 Conclusion

The present study demonstrates the strong cytotoxic potential of the *Sarcococca saligna* (SL) extract (SL-01) and its ethyl acetate fraction (SL-03) against DLD-1, HT-29, A549, and MCF-7 human cancer cell lines. Phytochemical profiling (LC-ESI-QTOF-MS/MS) identified eight steroidal alkaloids, of which two compounds, sarcorine C and salonine C, were isolated and structurally characterized. Both alkaloids exhibited selective cytotoxicity against HT-29 colon cancer cells while showing negligible toxicity toward noncancerous VERO cells, indicating a superior safety profile compared with FDA-approved drugs such as cisplatin and doxorubicin. Molecular docking revealed strong interactions of sarcorine C and salonine C with key cancer-associated proteins (CDK2, KRAS, CYP17A1, Bcl-2, MMP-2, and NOS). Extended 200 ns molecular dynamics simulations and MM-GBSA free energy calculations further validated these interactions, confirming stable protein–ligand complexes, favorable binding free energies (ΔG_bind = −42.6 kcal·mol^–1^ for sarcorine C, −37.4 kcal·mol^–1^ for salonine C), and robust structural stability. Sarcorine C displayed a particularly strong affinity for CDK2, while the electrophilic regions of salonine C may contribute to its predicted interactions with KRAS, suggesting a possible mechanism.

Importantly, both compounds demonstrated favorable ADMET properties and electronic features from DFT analyses, supporting their potential as orally bioavailable and low-toxicity therapeutic candidates. To the best of our knowledge, this is the first report of sarcorine C and salonine C as selective cytotoxic agents against HT-29 colon cancer cells. Overall, these findings position sarcorine C and salonine C as promising steroidal alkaloid leads for the preclinical development of colon cancer. Future in vivo studies will be crucial to validate their therapeutic potential, elucidate mechanisms of action, and advance them toward targeted, safer, and more effective anticancer therapies. This work eventually highlights *Sarcococca saligna* as a valuable medicinal plant, underscoring its potential as a sustainable source of bioactive steroidal alkaloids for anticancer drug discovery.

## 4 Experimental Section

### 4.1 Plant Material

Aerial parts of *S. saligna* were collected from Dunagiri Forest (Almora), Uttarakhand, India, located between 29° 36′ north latitude and 79° 30′ east longitude. The plant material was authenticated by senior author K. R. Arya. A voucher specimen, KRA-24498, was submitted to the departmental herbarium, CSIR-CDRI, Lucknow, India.

### 4.2 Plant Extraction

The collected plant material (SL) was air-dried and powdered. For the solvent extraction procedure, the dried and powdered plant material (890 g) was placed in a glass percolator, soaked in 95% ethanol, and kept at room temperature for 24 h. The extraction procedure was repeated four times, and the resulting percolated extract was collected, combined, and filtered.^62^ The combined filtrate was concentrated to dryness using a rotary evaporator at 40 °C. As a result, a dried ethanolic crude extract (SL-01) was obtained.

### 4.3 Fractionation of Ethanolic Extract

To separate the compounds from SL-01, on the basis of polarity, the SL-01 extract was further subjected to successive solvent fractionation to yield different solvent fractions.^62^ Hexane extraction yielded a hexane-soluble fraction (SL-04) and a hexane-insoluble residue. The hexane-insoluble residue was further extracted with ethyl acetate, producing an ethyl acetate-soluble fraction (SL-03) and an ethyl acetate-insoluble residue. This residue was suspended in water and extracted with *n*-butanol to obtain the *n*-butanol-soluble fraction (SL-02) and the water-soluble fraction (SL-05). All the solvent fractions were concentrated under reduced pressure using a rotary evaporator at 40 °C to give dried fractions SL-02, SL-03, SL-04, and SL-05 (Figure 17). The obtained extracts were weighed, and their percentage yields were calculated. These were kept at 4 °C until further use. These extracts and fractions were further evaluated for phytochemical and biological analysis.

### 4.4 Biological Assay

#### 4.4.1 Cytotoxicity Assay

The cytotoxicity of the SL-01 extract and its fractions SL-02, SL-03, SL-04, and SL-05 was determined using the standard colorimetric SRB (sulforhodamine B) assay for the measurement of cell viability, as described earlier.^62–66^ The SRB assay was selected owing to its highly sensitive, cost-effective, and reproducible approach for assessing cytotoxicity in different cell types.^63^ It provides quantitative data on cell viability, making it suitable for drug discovery, cancer research, and toxicology studies. Its nonradioactive nature and long-term storage capability add to its practicality and versatility, making it one of the best approaches for evaluating cytotoxic effects in research.^64^ Moreover, VERO cells were used as a control, while the standard anticancer drug doxorubicin (10 μM) was used as a positive control.^67^ All the fractions were dissolved in cell culture-grade dimethyl sulfoxide (DMSO) at a 100 mg/mL stock concentration. For each sample, triplicate wells were included.^62^ Initially, the cytotoxicity of the SL-01 extract and its fractions SL-02, SL-03, SL-04, and SL-05 was evaluated at a 100 μg/mL concentration for 48 h on a panel of 4 human cancer cell lines using the SRB assay. Based on the highest percentage inhibition of cancer cell growth, the most responsive antiproliferative fraction and its isolated compounds were further evaluated at concentrations ranging from 6.25 μg/mL to 100 μg/mL to obtain the IC_50_ values. The IC_50_ values were calculated by plotting a curve of cell number versus the log of drug concentration and using formulas described in the relevant literature,^62–65^ while the percentage (%) cell growth inhibition was calculated as percent cell growth inhibition (GI) using the following formula.^65^

$$\mathrm{GI}=[100-\left(\frac{absorbance\;of\;treated\;cells}{absorbance\;of\;vehicle\mathrm{‐t}reated\;cells}\right)\times 100$$

where vehicle control was the cells treated with DMSO only.

#### 4.4.2 Cell Culture

All human cancer cell lines, namely DLD-1, HT-29 (colon), A-549 (lung), and MCF-7 (breast), as well as the noncancerous cell lines i.e., VERO (monkey kidney fibroblast), were obtained from the American Type Culture Collection (ATCC) and revived from early-passage liquid nitrogen vapor stocks as required.^68^ The human cancer cell lines A-549, DLD-1, HT-29, MCF-7, and VERO were grown in the recommended media with 10% FBS. Cells were routinely inspected microscopically for a stable phenotype. The enumerated cells were dispensed into a 96-well tissue culture plate. Each well received 100 μL of the cell suspension containing 10,000–30,000 cells (depending on the cell line). The cells were then incubated at 37 °C in 5% CO_2_ concentration for 24 h before the addition of the test samples/standard drugs.^65^

### 4.5 Mass Spectrometric and Chromatographic Conditions

Liquid chromatography–electrospray ionization–quadrupole time-of-flight–tandem mass spectrometry (LC-ESI-QTOF-MS/MS) was employed for spectral analysis of the crude extract, its fractions, and the isolated compounds to ensure accurate identification and structural characterization

#### 4.5.1 LC-ESI-QTOF-MS/MS Analysis

LC-ESI-QTOF-MS/MS analysis comprised Agilent 6520 QTOF-MS coupled with Agilent 1200 HPLC equipped with an ESI source. The Poroshell 120 EC C18 column (50 mm × 4.6 mm, 2.7 μM) was used for HPLC separation.^62^ The mobile phase consisted of a mixture of 0.1% formic acid aqueous solution (A) and acetonitrile (B). The gradient elution was planned as follows: 25 to 37% (B) for the initial 5 min, then 37% (B) from 5 to 10 min, 37–40% (B) from 10 to 15 min, 40–25% (B) from 15 to 18 min, 25% (B) from 18 to 20 min. The flow rate was standardized to 0.4 mL/min, and the sample injection volume was 1 μL.

#### 4.5.2 Column Chromatography and NMR Characterization

The most active ethyl acetate fraction *S. saligna* was chromatographed on silica gel (100–200 mesh) for column chromatography (7 cm × 60 cm) using MeOH:chloroform solvent mixtures of increasing polarity. Elution was initiated with 100% chloroform, followed by stepwise gradients of methanol in chloroform (v/v) at 2%, 3%, 5%, 7%, 10%, 20%, and 50%. The eluates were collected as subfractions. To minimize irreversible adsorption of alkaloids onto silica, 1–2% diethylamine was added to all solvent mixtures. Subfractions were monitored by TLC and visualized with Dragendorff’s reagent, and subfractions with similar TLC bands were pooled.^69^ Repeated chromatography of the pooled subfractions eluted at 5% and 10% MeOH in chloroform yielded two pure steroidal alkaloidal compounds. Structure characterization was performed by mass spectrometry and ^13^C and ^1^H NMR using CDCl_3_ as a solvent and tetramethylsilane (TMS) as an internal standard. The frequency of the NMR instrument used was 500 MHz. The proton operating frequency was 500 MHz, and for carbon, it was 125 MHz.

### 4.6 In Silico Studies

#### 4.6.1 Ligand–Protein Preparation and Docking Analysis

The 3D structures of eight bioactive compounds identified through LC-MS analysis, including the isolated compounds sarcorine C (CID: 11373632) and salonine C (CID: 44358179), were retrieved from the PubChem database in Protein Data Bank (PDB) format.^33^ Screening of the receptor structures was conducted using the AutoDock molecular graphics system.^34^ Based on a comprehensive literature review and analysis of crystal structures of proteins that act as critical regulators in apoptosis and cell cycle regulation, six key target proteins associated with HT-29 colon cancer cells were selected: CDK2, CYP17A1, Bcl-2, MMP-2, NOS, and KRAS.^38–42^ These targets were chosen due to their relevance in colorectal and HT-29 cancer biology. CDK2 regulates cell cycle progression and is frequently dysregulated in colon tumors;^38^ KRAS mutations drive colorectal cancer progression;^50^ MMP-2 promotes invasion and metastasis;^70^ Bcl-2 overexpression confers resistance to apoptosis;^40^ CYP17A1 contributes to hormone-mediated tumor microenvironment regulation;^39^ and NOS isoforms influence angiogenesis and tumor growth.^42^

The three-dimensional structures of the target proteins were obtained in PDB format.^47^All nonprotein molecules, including water and other heteroatoms, were removed using **AutoDock Tools** to prepare the protein structures for docking.^34^ To optimize the structures of the ligands (bioactive compounds), energy minimization was carried out using the MMFF94 force field in Avogadro,^35^ ensuring that the ligands adopted optimal geometries for subsequent docking studies. Docking grids were defined around the active sites of each protein. For CDK2 (2A4L): center (*x* = 32.1, *y* = 25.4, *z* = 28.7 Å), dimensions 40 × 40 × 40 Å. For KRAS (4LPK): center (*x* = 15.2, *y* = −8.5, *z* = 20.1 Å), dimensions 40 × 40 × 40 Å. For CYP17A1 (3RUK): center (*x* = −10.3, *y* = 18.2, *z* = 45.7 Å), dimensions 40 × 40 × 40 Å. For MMP-2 (1HOV): center (*x* = 38.6, *y* = 22.4, *z* = 30.9 Å), dimensions 40 × 40 × 40 Å. For NOS (3E7G): center (*x* = 5.3, *y* = 17.9, *z* = 42.5 Å), dimensions 40 × 40 × 40 Å. Exhaustiveness was set to 8 for all dockings. The eight bioactive compounds were then docked into the prepared protein structures using AutoDock Vina.^71–74^ The docking results were subsequently analyzed to calculate key parameters such as the inhibition constant, binding energy, and ligand efficiency.^55,56,74^ Upon completion of the docking process, the compounds exhibiting the lowest binding affinity (kcal/mol) and the best conformations were selected for further analysis. The key binding interactions including hydrogen bonds and hydrophobic interactions were observed using Discovery Studio Visualizer, with particular emphasis on the residues involved in these interactions.^37^ The binding site of CDK2 was defined around residues Asp86 and Gln131, while the KRAS binding site was defined around Gly12 and Gly13. A grid box covered the entire binding pocket for both targets, and the exhaustiveness was set to 8.

#### 4.6.2 Molecular Dynamics Simulations

Molecular dynamics (MD) simulations were performed to validate the stability and binding characteristics of sarcorine C and salonine C with CDK2 (PDB ID: 2A4L), alongside the reference inhibitor roscovitine and apo CDK2. Each system was solvated in a TIP3P cubic box (10 Å buffer), neutralized with 0.15 M NaCl, and energy-minimized (50,000 steps, steepest descent). After equilibration (NVT: 200 ps, NPT: 1 ns), production MD simulations were conducted for **200 ns** using GROMACS 2022.4 with a 2 fs time step, PME electrostatics, and LINCS constraints. Trajectories were analyzed for RMSD, RMSF, hydrogen bonds, ***R*_g_**, SASA, and MM-GBSA free energies.^52,72^

#### 4.6.3 ADMET Analysis and Drug-Likeness

Studies of ADMET (absorption, distribution, metabolism, excretion, and toxicity) evaluate properties that are important and useful in the development of new natural compounds with improved pharmacokinetic and pharmacodynamic properties.^57,64^ Therefore, compounds with the best binding affinity were further evaluated through in silico ADMET studies. Key parameters analyzed included mutagenicity, cytotoxicity, blood–brain barrier absorption, water solubility, liver toxicity, and gut absorption. SwissADME, the vNN process, and MolSoft were used to study the ADMET properties of the screened bioactive compounds. Pharmacodynamic properties of effective ligands were predicted using the Molinspiration online server.^58^

#### 4.6.4 DFT and MESP Calculations

DFT calculations were performed in **Gaussian 16**, utilizing the B3LYP functional with the **6-31G­(d)** basis set. Geometry optimization yielded stable conformers for both ligands. MESP calculations were then performed to analyze electron distribution and identify potential nucleophilic and electrophilic sites.^71–74^

### 4.7 Statistical Analysis

Statistical analysis was performed using GraphPad Prism. The *in vitro* experiment results were expressed as the mean ± standard deviation (mean ± SD) of 3 replicates. Significant differences are represented with a *p*-value in appropriate graphs, calculated by Student’s *t* test.

## Supplementary Material



## Notes

^1^H and ^13^C NMR spectra, as well as HMBC, HSQC, and COSY spectra of salonine C and sarcorine C (PDF)

## Abbreviations

A-549: Lung carcinoma
BPC: base peak chromatogram
CC: column chromatography
DLD-1: colorectal adenocarcinoma
DMSO: dimethyl sulfoxide
DOX: doxorubicin
FBS: fetal bovine serum
HT-29: colorectal adenocarcinoma
IC_50_: half-maximal inhibitory concentration
MCF-7: breast cancer cell lines
SRB Assay: sulforhodamine B assay
TLC: thin-layer chromatography
VERO: noncancerous monkey kidney fibroblast cell lines
PDB: Protein Data Bank
MD: molecular dynamics
