# Supplementary material for: Steroidal Alkaloids from Sarcococca saligna (Buxaceae): In Vitro and In Silico Evaluation of Their Cytotoxic Potential
Source: ACS Omega. 2025 Oct 10;10(41):48111–29. doi: 10.1021/acsomega.5c04143 (PMC12547747; doi:10.1021/acsomega.5c04143)
Supplement: Supplementary file 1 [file ao5c04143_si_001.pdf]

Steroidal Alkaloids from *Sarcococca saligna* (Buxaceae): In Vitro and In Silico Evaluation of Their Cytotoxic Potential

Neha Sahu[ad]*, *Amit Dubey*[b], *Nitesh Singh*[c], *K.R. Arya*[d], *Pragya Yadav*[e], Priyank Chaturvedi[f], *Sanjeev Meena*[f], *Vijaya Shukla*[g], *Dipak Datta*[f], *T. Narender*[e], *Brijesh Kumar*[g] *Bikash Kumar Rajak*[h]

[a]Department of Botany, University of Lucknow – 226031, Uttar Pradesh, India.
[b]Center for Global health Research, Saveetha Medical College and Hospitals, Saveetha Institute of Medical and Technical Sciences, Chennai - 600077, Tamil Nadu, India.
[c]Faculty of Agricultural Sciences, SGT University, Gurugram – 122505, Haryana, India.
[d] Botany division, [e]Medicinal and Process Chemistry Division, [f]Biochemistry, [g]Sophisticated Analytical Instrument Facilities, CSIR-Central Drug Research Institute, Lucknow 226031 (U.P.) India.
[h]Department of Bioinformatics, School of Earth, Biological and Environmental Sciences, Central University of South Bihar, Gaya – 824236, Bihar, India.

List of authors and their email address

*Neha Sahu, sahu_neha@lkouniv.ac.in
Amit Dubey, amitdubey@saveetha.com
Nitesh Singh, niteshigntu@gmail.com
K.R. Arya, aryakr@rediffmail.com
Pragya Yadav, pragyayadav8918@gmail.com
Priyank Chaturvedi, priyankcdri@gmail.com
Sanjeev Meena, sanjeev.meena@cdri.res.in
Vijaya Shukla, vijayavaidehyshukla@gmail.com
Dipak Datta, dipak.datta@cdri.res.in
T. Narender, t_narendra@cdri.res.in
Brijesh Kumar, brijesh_kumar@cdri.res.in
Bikash Kumar Rajak, bkrbikash0@gmail.com

***Corresponding author:**
Dr. Neha Sahu
Assistant Professor, Department of Botany,
University of Lucknow
Lucknow, U.P. 226031
Contact no: +917007087014
E-mail address: sahu_neha@lkouniv.ac.in
ORCID ID: 0000-0001-7478-5873

Supplementary images of NMR

<u>NMR data of Salonine C and Sarcorine C</u>

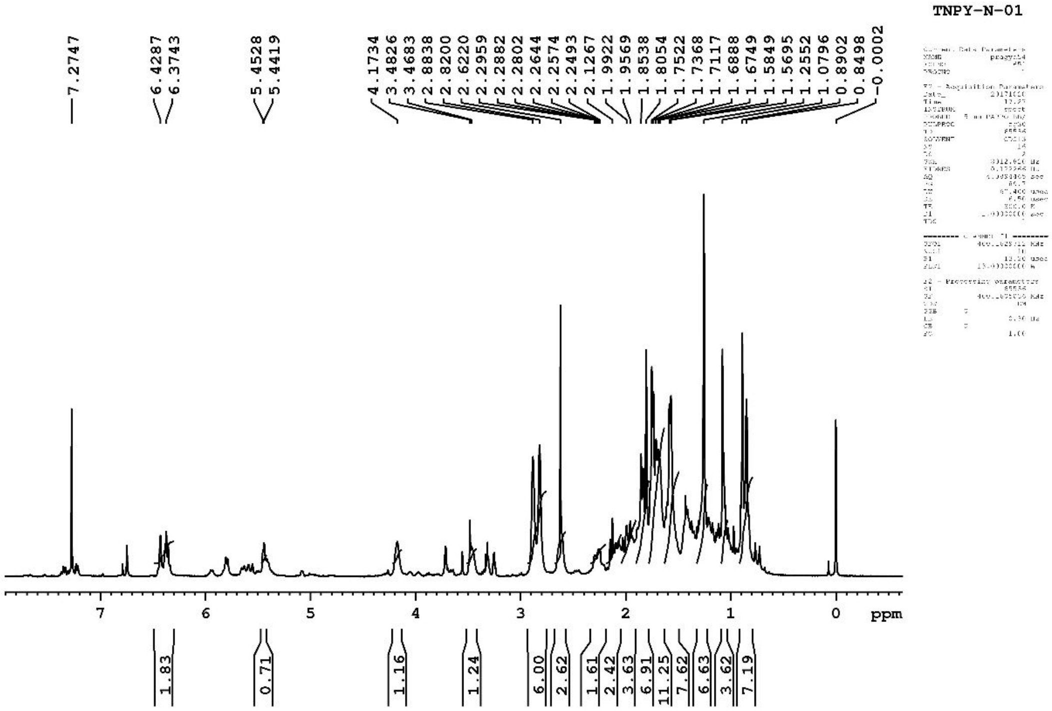

[1] H NMR of Salonine C

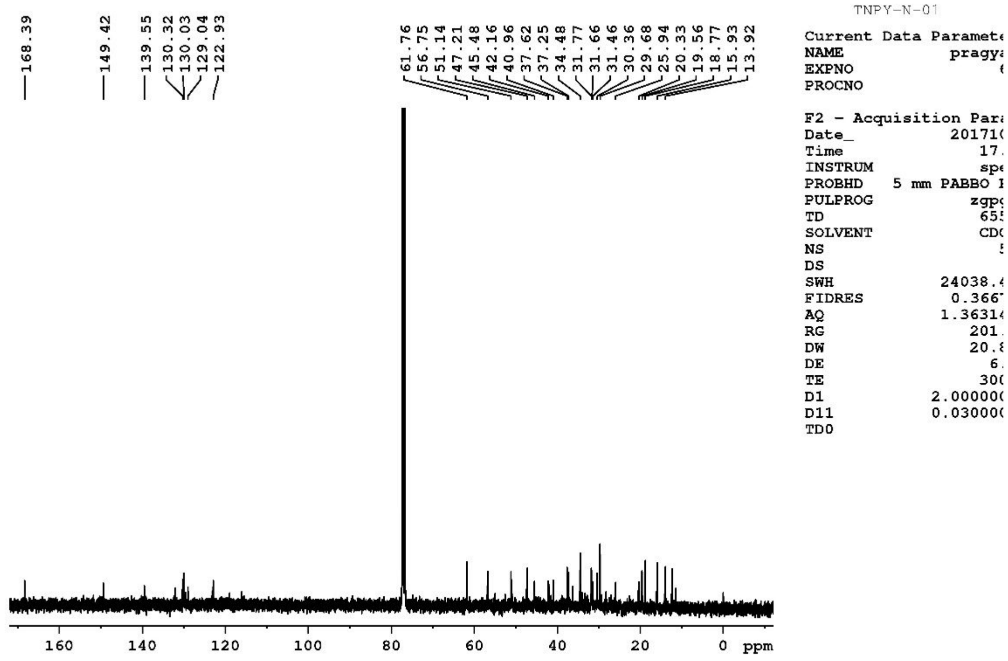

^{13}C NMR of Salonine C

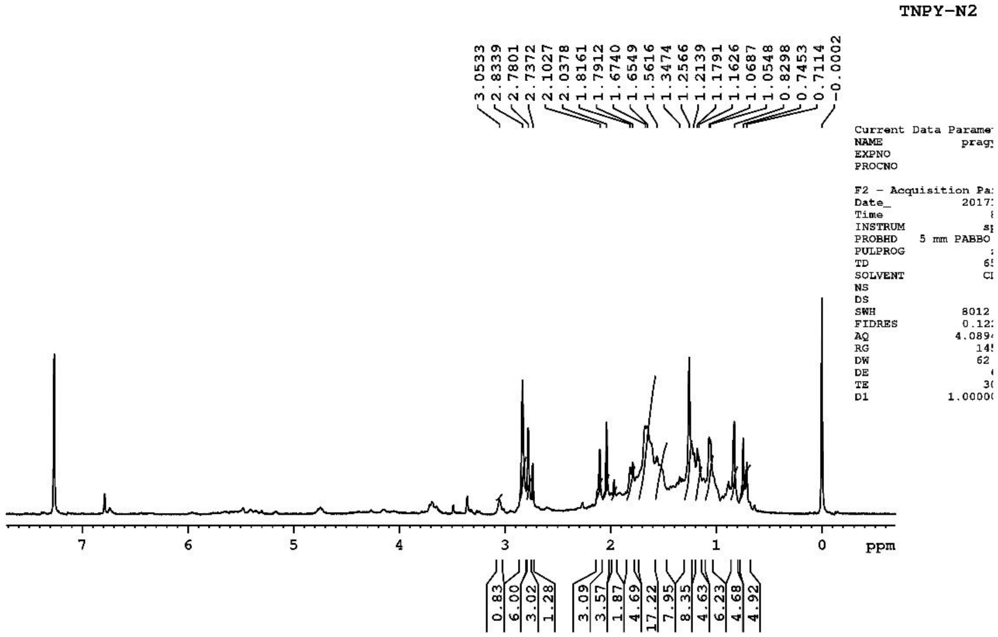

^{1}H NMR of Sarcorine C

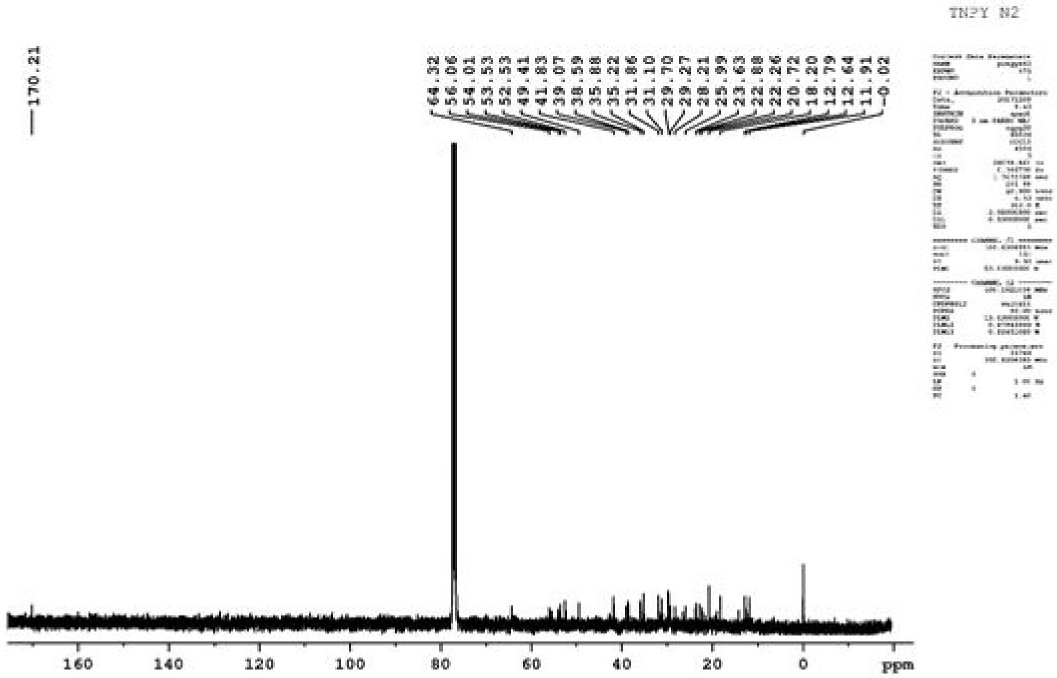

^{13}C NMR of Sarcorine C

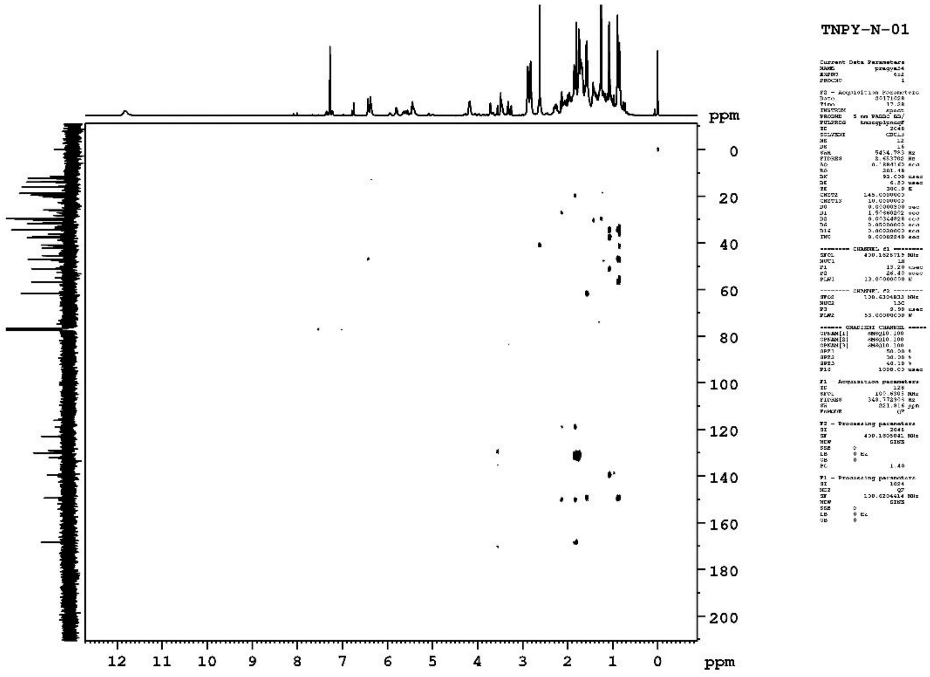

HMBC of Salonine C

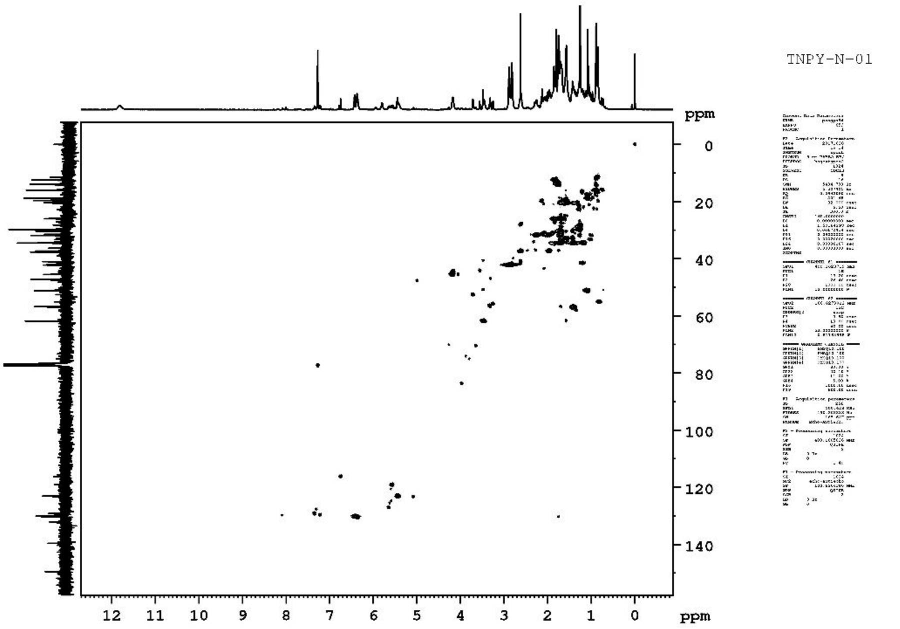

HSQC of Salonine C

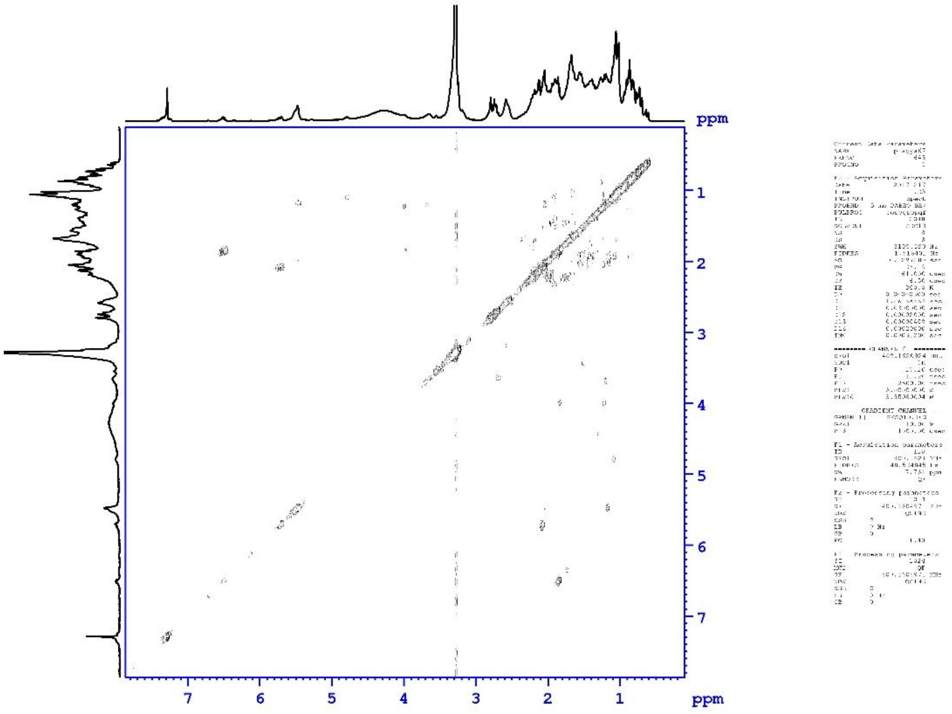

COSY of Salonine C

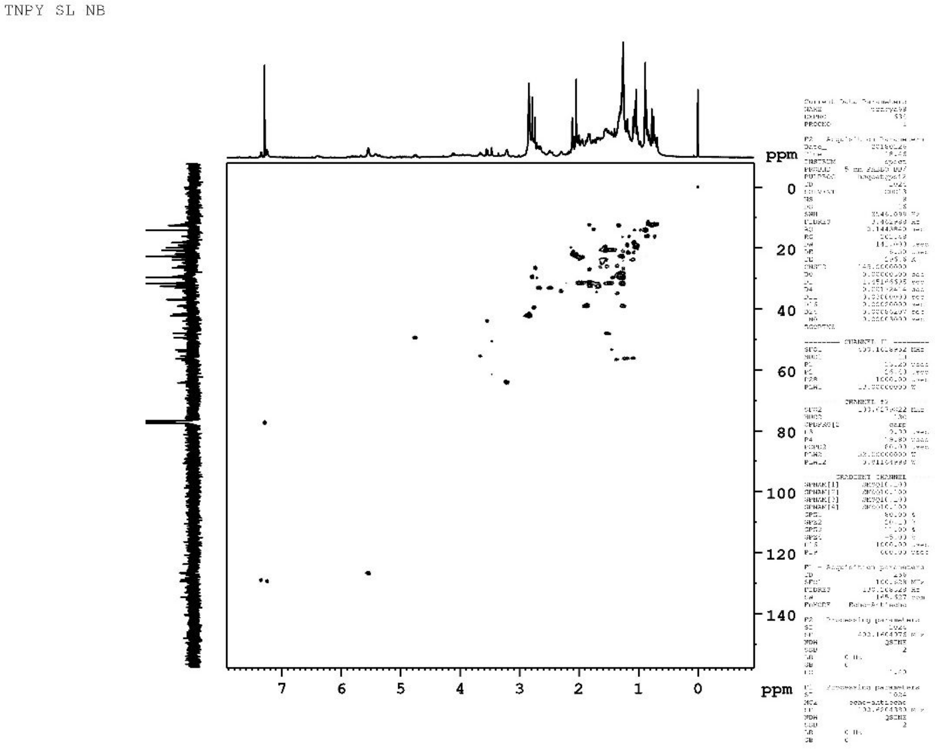

HMBC of Sarcorine C

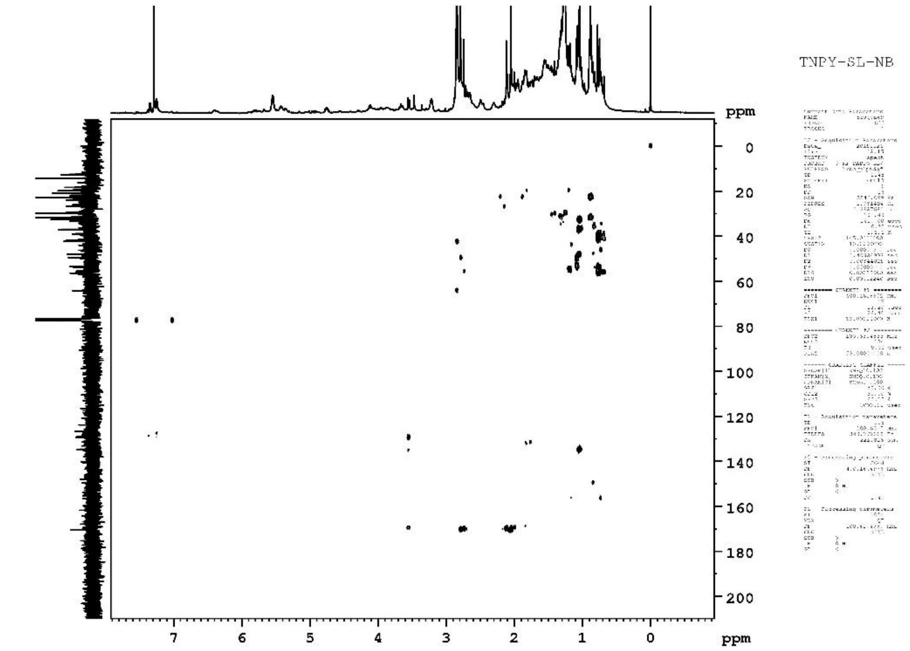

HSQC of Sarcorine C

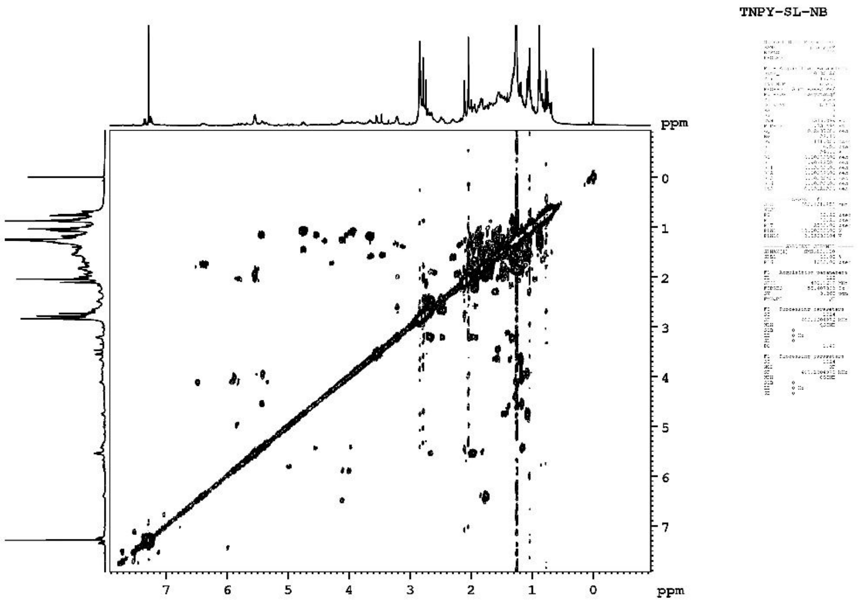

COSY of Sarcorine C
